# More Than a Stick in the Mud: Eelgrass Leaf and Root Bacterial Communities Are Distinct From Those on Physical Mimics

**DOI:** 10.1111/1758-2229.70086

**Published:** 2025-04-30

**Authors:** Melissa R. Kardish, John J. Stachowicz

**Affiliations:** ^1^ Department of Evolution and Ecology University of California Davis California USA; ^2^ Center for Population Biology, University of California Davis California USA; ^3^ Center for Bio/Molecular Science and Engineering, US Naval Research Laboratory Washington, DC USA

**Keywords:** community assembly, microbe:higher organism interactions, microbial communities, microbial ecology

## Abstract

We examine the role of physical structure versus biotic interactions in structuring host‐associated microbial communities on a marine angiosperm, *Zostera marina* , eelgrass. Across several months and sites, we compared microbiomes on physical mimics of eelgrass roots and leaves to those on intact plants. We find large, consistent differences in the microbiome of mimics and plants, especially on roots, but also on leaves. Key taxa that are more abundant on leaves have been associated with microalgal and macroalgal disease and merit further investigation to determine their role in mediating plant–microalgal–pathogen interactions. Root associated taxa were associated with sulphur and nitrogen cycling, potentially ameliorating environmental stresses for the plant. Our work identifies targets for future work on the functional role of the seagrass microbiome in promoting the success of these angiosperms in the sea through identifying components of microbial communities that are specific to seagrasses.

## Graphical abstract

Unique communities inhabit eelgrass leaves and roots compared to physical mimics and their environment. We examine which taxa vary to determine what host–microbe relationships may be mediate by biotic interactions. This indicates that eelgrass leaves and roots may just unique environments for microbes, but also could indicate more active partnership/inhibition involved in the host–microbe relationship worthy of further exploration.

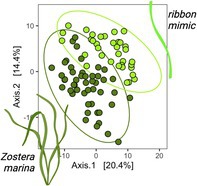

## 1 Introduction

The role of microbiomes in host ecology is increasingly recognised as an important force driving the ecology and dynamics of a broad range of ecosystems. Host–microbe interactions range in strength and direction (McFall‐Ngai et al. 2013; Hammer et al. 2019) with microbes providing net benefits to their hosts in some cases, parasitizing hosts or other members of the microbial community in others, and participating in many symbiotic relationships in between these extremes (Trivedi et al. 2020). Yet hundreds or thousands of microbial taxa associate with any given host, and we generally know little about the extent to which most associations rely on specific host traits such as morphology, physiology or metabolites. In most cases, our knowledge is limited to comparing host‐associated microbes with a larger environmental pool, such as soils or seawater, and identifying taxa over‐represented on hosts compared to the environment (Knights et al. 2011; Fahimipour et al. 2017; Xiong et al. 2021). These approaches often identify hundreds of taxa positively associated with hosts, still leaving a major challenge for developing an understanding of the extent to which particular microbes interact closely with, and impact, hosts. However, this approach does not distinguish the role of the provision of physical structure versus host‐specific biology; incorporating this level of distinction would identify taxa that require not just the structure but the presence of a living host and therefore a greater potential for reciprocal interactions with the host. One way to distinguish the relative importance of physical structure from living organisms is to use physical mimics to assess how microbial communities develop differently in the absence of biotic interactions with the host.

Mimicking environments to learn more about host–microbe interactions—whether through simple physical models, reconstituting biochemical environments, or even using germ‐free organisms—has been used across a diversity of taxa to assess critical members microbial communities as well as to assess how the overall structure of their communities vary under stress. This addresses an important forefront of host–microbiome research—establishing how those interactions form and are stabilised (Trevathan‐Tackett et al. 2019). For example, in terrestrial plants, finely mimicked leaf surfaces have led to insights on where *Escherichia coli* resides on spinach leaves based on water retention on structural mimics (Zhang et al. 2014) and an artificial human gut has been used to demonstrate interactions between anti‐inflammatory bacteria and epithelial cells (Zhang et al. 2021). In addition to identifying how an organism interacts with the microbes, mimics can also be used to describe specific assembly patterns that do not occur without the biological host environment or identify key partners that occur at higher abundances in a host environment (e.g., Lee et al. 2019). For example, with a structural mimic, the differences observed could emphasise how hosts and microbes interact outside of simply that structure and lead to more insights about host‐tissues, immune responses, or exudates specifically effect host–microbe interactions.

Host‐associated microbial communities have been identified across organisms to assemble in distinct non‐random ways though the degree of specificity varies (Taylor et al. 2004; Ambika Manirajan et al. 2016). Microbial associates can be highly specific (e.g., the bobtail squid and *Vibrio fisherii*; McFall‐Ngai and Ruby 1991), might be based on functions of microbes (e.g., on *Ulva australis* where communities share functional genes rather than taxonomic composition; Burke et al. 2011), or might be transitory/happenstance associations (e.g., high numbers of soil bacteria in Lycaenid butterfly gut microbiomes; Whitaker et al. 2016). In addition, a host can interact with a microbiome with different levels of restrictiveness: a host might provide a highly restricted environment that could or could not be heavily‐modified by a host (e.g., a gut microbiome; Garland et al. 1982; Rinninella et al. 2019) or a less restrictive environment where even with environmental modification from the host many microbes could enter the community (e.g., skin microbiome; Byrd et al. 2018). Distinguishing among these types of microbial communities may offer insight into the intensity of interactions with a host. For instance, if we distinguish that a less restrictive surface had a microbiome unassociated specifically with a host, we might infer limited direct unique interactions with that host.

We investigate the role of physical structure versus biotic interactions in structuring the surface microbiomes of seagrass, specifically, eelgrass, *Zostera marina* . Eelgrass is a marine flowering plant; its roots and rhizomes grow in highly sulphidic sediment and its leaves, while primarily exposed to seawater, can be periodically exposed to air at low tides (Jørgensen 1982). Seagrass leaves are distinct from terrestrial angiosperms in several ways, including absence of stomata as well as primary exposure to seawater rather than air (Olsen et al. 2016). Our previous work showed broad overlap in the composition of leaf and water microbiomes (Fahimipour et al. 2017), but did find some microbes preferentially associated with leaves. Comparison of microbiomes among species of seagrass that grow in the same environment show that some harbour distinct microbial communities on their leaves from other species (Garcias‐Bonet et al. 2021) while some seagrass species have broad overlap in their microbiomes (Ugarelli et al. 2017; Kaimenyi et al. 2018). At least some microbial taxa are disproportionately found on seagrass compared to water, though it is not clear the extent to which leaf microbiomes differ from those that accumulate on inert surfaces in marine systems, where biofilms develop on surfaces at a fast rate (Fischer et al. 2014). Mimicked seagrass leaves have long been used to investigate community structures and show similar macroinvertebrate (Healey and Hovel 2004), fish (Bell et al. 1985) and microalgal communities (Horner 1987; Pinckney and Micheli 1998) to natural seagrass and provide an obvious approach for distinguishing substrate generalists from seagrass‐specific associates. We adopt this approach to narrow the microbiome of seagrass leaves from the pool of over a hundred of taxa known to be enriched on leaves relative to surrounding seawater to a smaller comparison of those that attach to similar surfaces.

Similarly, root surfaces have bacterial communities distinct from adjacent sediments (Fahimipour et al. 2017), but the influence of physical structure versus host biology on root microbiome composition is not yet clear. Roots inhabit anoxic and highly sulphidic sediments that without mitigation can lead to sulphide intrusion decreasing plant growth and health (Hasler‐Sheetal and Holmer 2015). Various mechanisms exist to mitigate this environment and reduce sulphide intrusion into the plant including radial oxygen loss (ROL) from growing roots (Pedersen et al. 2004), direct partnerships with sulphide‐oxidising bacteria (Smith et al. 2004), and three‐way symbiosis with lucinid clams hosting sulphide‐oxidising bacteria (van der Heide et al. 2012; de Fouw et al. 2018). Since direct association of seagrass with sulphur oxidisers is known (Fahimipour et al. 2017) and given the leak of oxygen and sugars out of the roots (Sogin et al. 2022), it seems likely that the plant plays an important role in root microbiome assembly. Examining mimicked root environments is less common in seagrass than use of mimicked leaves and previous work has focused on sediment stabilisation processes (Temmink et al. 2020) rather than influence on biotic community structure.

Here, we explicitly test whether seagrass roots and leaves assemble microbiomes that are distinct from physical mimics at a range of sites and seasons within a harbour. By comparing live plants with biologically inactive surfaces mimicking some physical aspects of the host, we test explicitly whether seagrass cultivates a unique microbiome on its leaves and/or roots. Differences in the bacterial communities between physically mimicked environments and plants could indicate bacteria that might be either attracted by specific biological aspects of a plant or that might be selected by plants for biologically important roles. Thus, such an approach can identify the role of the plant in microbial assembly and hint at specific key processes, while also potentially identifying microbial partners that may play key functional roles in the seagrass microbiome as targets for future experimentation.

## 2 Results

**FIGURE 1:**
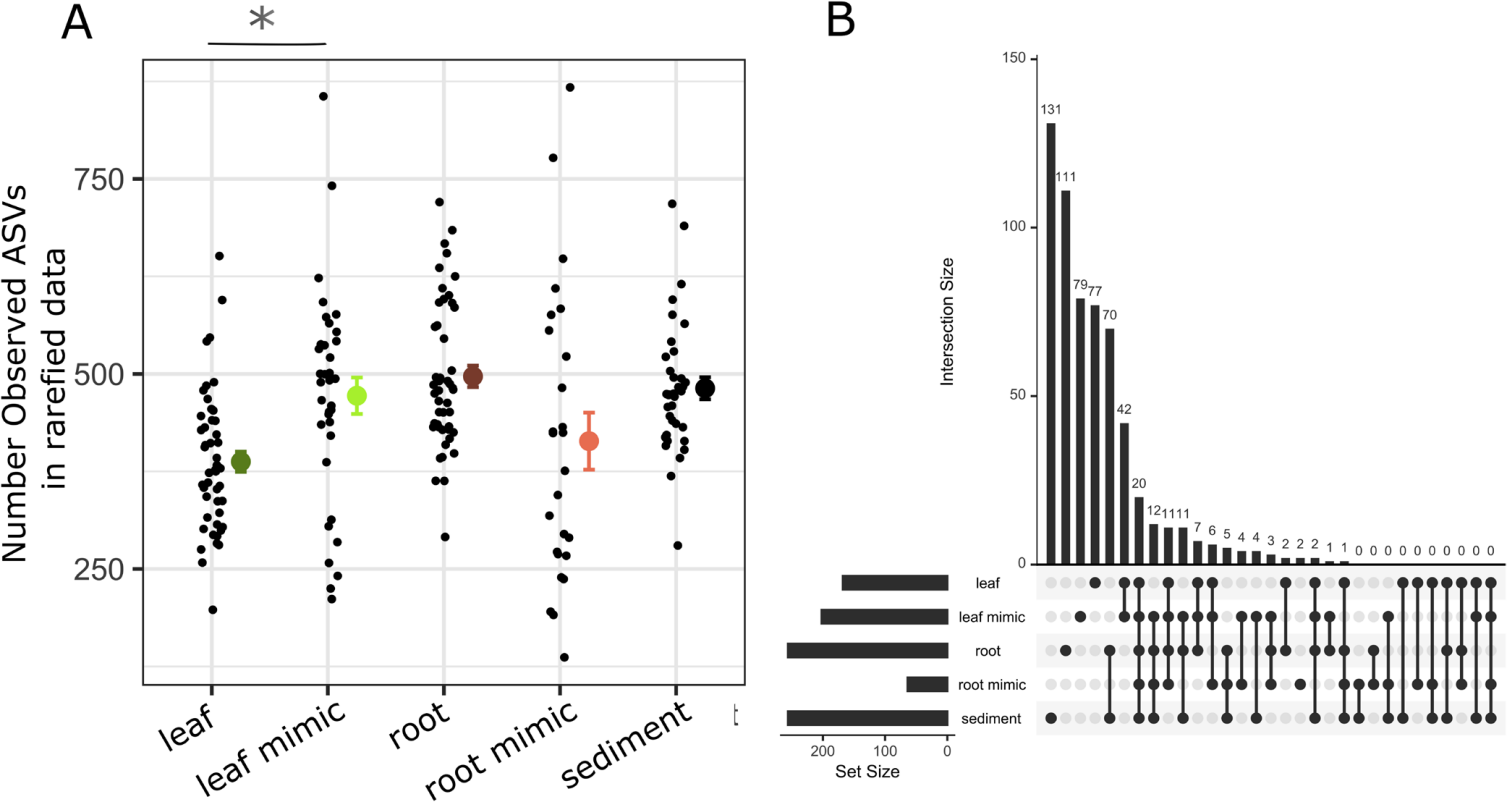
(A) Mean amplicon sequence variant (ASV) richness found in each sample type. Smaller black dots are raw data and larger dots with error bars are means and standard errors for each sample type. Leaf mimic bacterial communities had a higher mean richness than leaf bacterial communities (*p* < 0.001); root mimic, root, and sediment bacterial communities did not differ in mean community richness. (B) Overlap among core bacterial communities showing shared ASVs present in each sample type in at least 50% of samples at at least a 1% relative abundance. The diagram is a bar plot of shared community memberships, equivalent to a Venn diagram. The five rows on the bottom indicate the different categories of samples (same as in A). Set size indicates how many ASVs are found in that category (i.e., there are fewer unique ASVs in root mimics than in other categories). Across the x‐axis of the main figure is the intersection being compared in that bar. For example, there are 131 ASVs only found in sediment communities, and there are 70 ASVs only found in roots or sediments.

**FIGURE 2:**
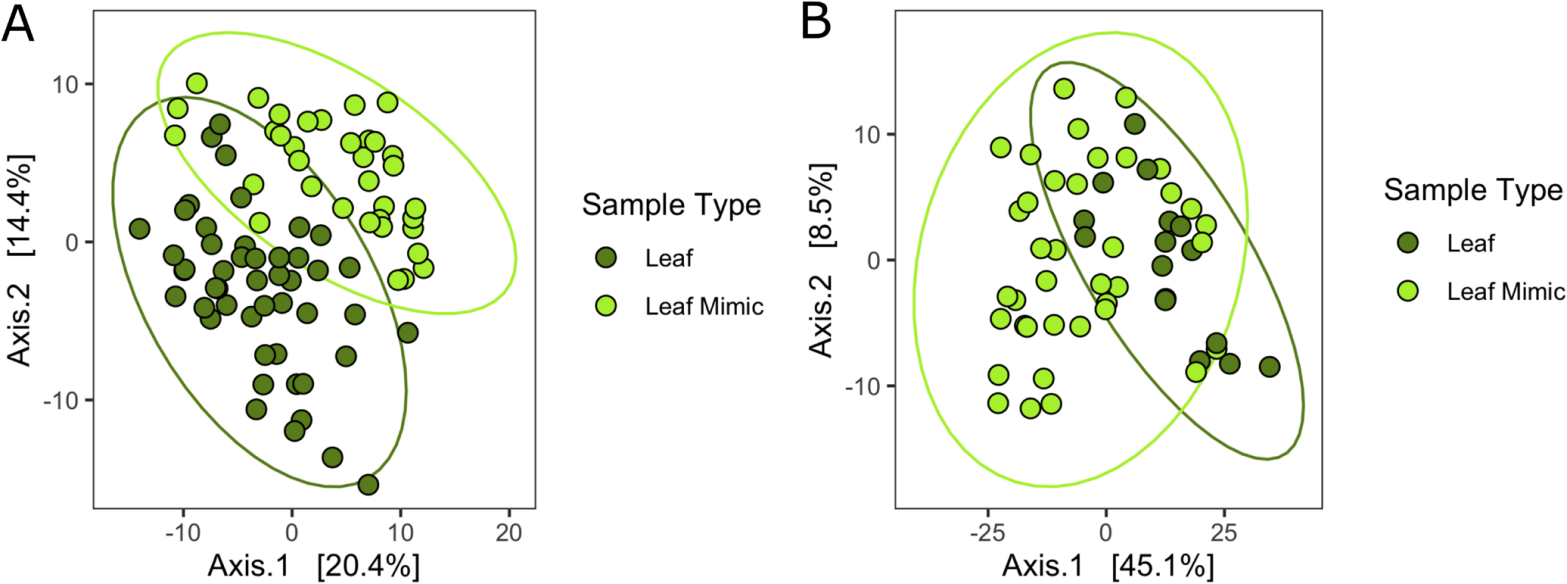
(A) Ordination of bacterial community structure based on principal coordinate analysis of phylogenetic‐isometric log‐ratio transformed distances. (B) Ordination of predicted Metacyc pathways structure based on principal coordinate analysis of centred log‐ratio transformed distances. Bright green points are communities on leaf mimics and dark green points are communities on leaves. Leaf and leaf mimic communities in both analyses are distinct from each other (PERMANOVA *p* < 0.001).

The bacterial assemblages associated with leaves and roots differed from those on their corresponding physical mimics in alpha diversity and/or community composition. However, the extent of this differentiation of eelgrass microbiomes from artificial substrates was stronger in roots than leaves (Figures 1 and 2). For example, leaves and mimics shared 86 taxa (42 of which were only found on leaves and mimics), whereas roots and mimics had 39 taxa shared between them, none of which were found only on these two substrates (Figure 1B). Roots had more unique taxa than leaves (111 vs. 77), and leaf mimics had similar number of unique taxa to leaves (79) whereas root mimics had only two unique taxa (Figure 1B). These differences among substrates were highly consistent across four sites and three time points (see results below), despite previously identified seasonal and site‐specific microbial compositional elements at these sites (Kardish and Stachowicz 2023), indicating a strong impact of live plants on the microbiome. Thus, we focus our presentation of results on the consistent effects of substrate across sites and time (each of which is controlled for in our statistical models). For an overview of sites data presented split by site and time point, see Figure S1.

### 2.1 Leaves

Leaf mimics had greater ASV richness than leaves (negative binomial glm with crossed random effects for month and site, estimate = 0.19738, standard error = 0.05802, *z*‐value 3.402, *p* = 0.0007; Figure 1A). The ASV composition of the leaf and mimic communities was different (PERMANOVA, *F* = 12.09, *p* = 0.001, *r* ^2^ = 0.13, Figure 2A), though there was no difference in variance among‐leaf versus among‐mimic communities (betadisper ANOVA, *p* = 0.73). When examining core ASVs (present in at least 50% of samples of a type at at least 1% detection rate), we found that roughly half the ASVs found on leaves were not found on any other substrate (77 of 168 ASVs; 46%) while most of the remaining were shared with those on leaf mimics (88 of 168, 52%). (Figure 1B). This degree of overlap in core taxa was the greatest of any pairwise comparison among sample types; The same patterns were present when we examined all ASVs rather than just the core (Figure S2). Predicted Metacyc pathways, based on a cross‐domain database of metabolic pathways and enzymes (Caspi et al. 2014), also differed between leaves and mimics (PERMANOVA, *r* ^2^ = 0.05488, *F* = 4.7035, *p* = 0.001), though this effect was weaker than for the sequence based compositional differences (Figure 2B).

To determine which components of the eelgrass leaf microbiome may be unique and involved in specific interactions with the non‐physical components of the eelgrass microbiome, we tested which ASVs were more abundant in leaves than mimics. Additionally, in Table 1, we identify which of these differentially abundant ASVs we did not detect in the other condition. We identified 140 ASVs were relatively more abundant on leaves and 101 were relatively more abundant on leaf mimics by using a likelihood ratio test comparing two negative binomial generalised liner models with and without ‘mimic versus non mimic’ as a factor implemented in DESEQ2. Only three families contained more than 10 ASVs that varied between mimics and leaves (Table 1): Flavobacteriaceae (10 higher on leaves, 17 higher on mimics), Rhodobacteraceae (31 higher on leaves, 20 higher on mimics), and Saprospiraceae (26 higher on leaves, notably only one higher on mimics). Within these families several genera were represented by multiple ASVs. These included *Kordia* (three ASVs higher on leaves, and notably none on mimics), *Ulvibacter* (two higher on leaves, three higher on mimics), *Octadecabacter* (one higher on leaves, one higher on mimics), *Sedimentitalea* (one higher on leaves, one on mimics), *Sulfitobacter* (two higher on leaves, two higher on mimics), *Tateyamaria* (one higher on leaves, one on mimics), *Yoonia‐Loktanella* (four higher on leaves), *Lewinella* (three higher on leaves), *Portibacter* (two higher on leaves), and *Rubidimonas* (three higher on leaves). Many other families contained fewer than 3 ASVs that varied between leaves and mimics (Tables 1 and S1), and within these 15 genera contained multiple ASVs that varied between leaves and mimics (Table S2). All taxa that varied can be found in Table S3.

**TABLE 1:** For both leaves and root communities, the five families (and the genera within them) that had the most ASVs that significantly varied between mimics and seagrass substrate based on differential expression analysis in DESeq2 and the numbers of individual ASVs that varied within those groups if more than one (in black).

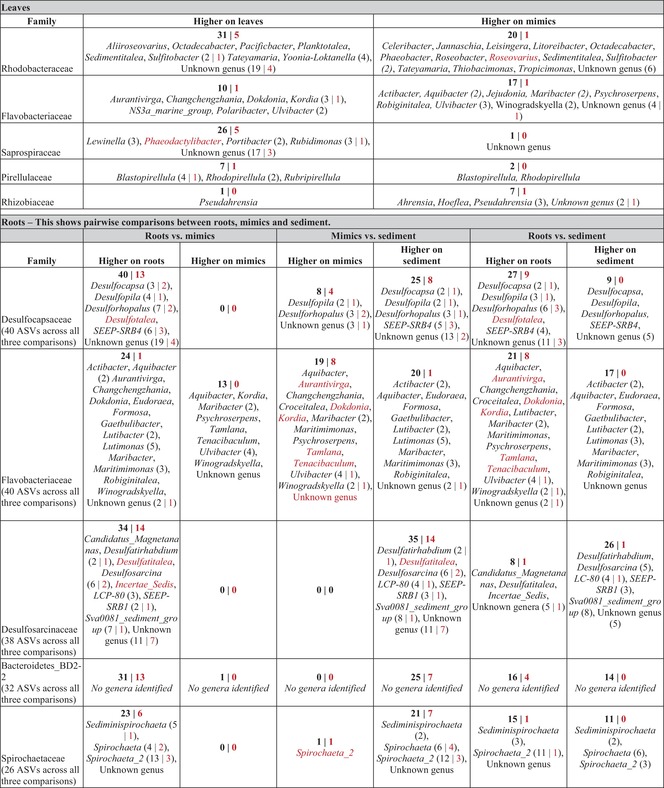

*Note:* The numbers in red indicate that number of ASVs where there was a difference in relative abundance by DESeq2 and there was no member found in the opposite category (e.g., 40 ASVs were present at a higher relative abundance in rots than mimics and 13 of these were not found on any mimic root). See Tables S1 and S6 for complete lists for leaves and roots respectively.

When we examined predicted pathways that changed between the leaf and leaf mimic microbiomes, we identified 53 pathways that changed, 16 upregulated in leaf microbiomes and 37 upregulated on mimic microbiomes (Table S4). These predicted pathways included differences in amino acid degradation, starch degradation, and denitrification. These predicted pathways did not always indicate expected differences between plants and mimic communities or produce clear candidate predicted pathways, likely at least in part due to limits in prediction of environmental microbial pathways, so we have focused on taxonomic differences.

### 2.2 Roots

**FIGURE 3:**
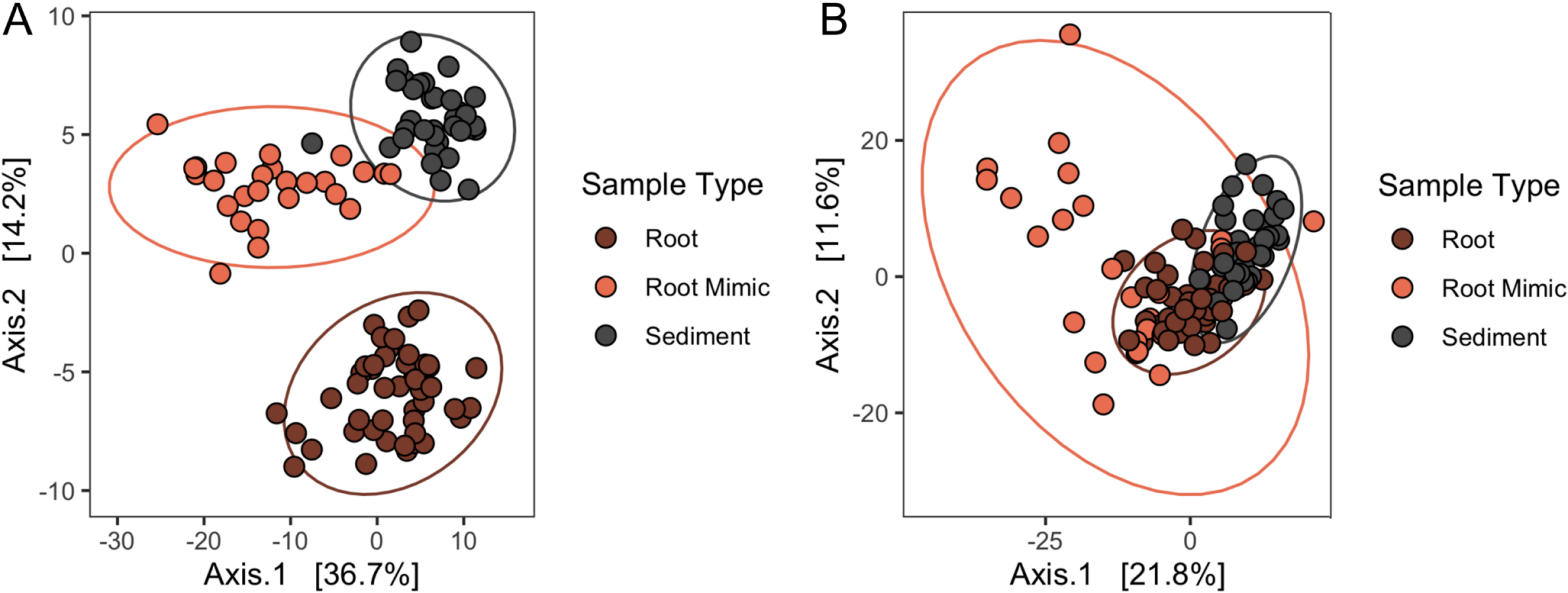
(A) Ordination of bacterial community structure based on principal coordinate analysis of phylogenetic‐isometric log‐ratio transformed distances. (B) Ordination of predicted Metacyc pathways structure based on principal coordinate analysis of centred log‐ratio transformed distances. Red‐orange points are communities on root mimics, dark brown points are communities on roots, and grey points are communities in sediments. All communities are distinct from each other in each analysis (PERMANOVA *p* < 0.001).

We found strong ASV compositional differences roots, mimics and sediment, but roots were more distinct from mimics and sediment than sediment and mimics were from each other (PERMANOVA *r* ^2^ = 0.3480 = 37.9015, *p* = 0.001, see Table S4 for pairwise comparisons). Roots and mimics did not differ in ASV richness (negative binomial glm with crossed random effects for site and month, *p* = 0.28, Figure 1A) or variance (betadisper ANOVA, *p* = 0.29). When including sediments in the comparisons, richness did not differ among the three (negative binomial glm with crossed random effects for site and month, *p* = 0.31) but microbiome variance among samples was less among sediment samples than either of the other two groups (betadisper ANOVA, *p* < 0.001, Tukey's HSD sediment vs. mimic *p* = 0.0001, vs. roots *p* = 0.004). When we examine overlap in predicted Metacyc pathways, we found that there was a significant difference between roots, sediments and mimics (PERMANOVA, *r* ^2^ = 0.18903, *F* = 12.534, *p* = 0.001), though this effect was weaker than for the sequence based compositional differences (Figure 3B, see Table S5 for pairwise differences).

When examining core ASVs (present in at least 50% of samples of a given type at at least 1% detection rate), we found that roots and sediments largely harboured distinct bacterial communities, by ASV (Figure 1B), though root mimics had few ASVs unique to its core microbiome (only 2 ASVs unique to root mimics); again, we saw the same patterns when including all ASVs in these analyses, and not just the core (Figure S2). Of 256 ASVs in the core root microbiome, 111 or 43% were found only on roots and 181 (71%) were found only on roots and in sediments. Only 52 core root ASVs (20%) were shared between roots and root mimics. When we examined all ASVs (without core restrictions), root mimics had more taxa unique to their sample type, indicating considerable variability in communities assembled on root mimics and a large contribution of rare ASVs (Figure S2).

We found many ASVs varied in abundance between these groups (457 between sediments and mimics, 505 between roots and sediments, and 486 between roots and mimics). Of these, the majority were at higher relative abundances on roots or sediments compared to mimics (comparing roots to mimics, 437 were higher on roots, and 49 were higher on mimics; comparing sediment to mimics, 359 were higher in sediments, 98 were higher on mimics; comparing roots to sediments 265 were higher on roots, 240 were higher in sediments; see Table 1 for families with the most representatives, Table S6 for more details and Table S7 for all ASVs that varied). The families that had the largest number of taxa vary among sample types included Spirochaetaceae (26 ASVs), Bactoroidetes BD2‐2 (32 ASVs), Desulfosarcinaceae (38 ASVs), Desusulfocapsaceae (40 ASVs), and Flavobacteriaceae (40 ASVs). Within the families Spirochaetaceae, Bactoroidetes, Desulfosarcinaceae, and Desusulfocapsaceae, most ASV were at greater relative abundance on roots than mimics (Table 1). In Flavobacteriaceae, roughly equal numbers of ASVs were more abundant in roots versus mimics versus sediment, sometimes within genus and sometimes between (Table 1). A few families showed a higher abundance of ASVs on roots compared to both sediments and mimics including: Desulfobacteraceae, Lachnospiraceae, Marinilabiliaceae, Moduliflexaceae, and Prolixibacteraceae (Table S6).

While the predicted pathways that varied were numerous and not particularly remarkable (as indicated in Table S8), we found that indicated pathways were generally indicated to be upregulated on mimics in pairwise comparisons (133 predicted pathways higher in mimics compared to 32 in sediments, and 137 higher on mimics compared to 35 on roots). Again, we anticipate that these results are due to limitations of predicting environmental microbial pathways (especially when finding lower numbers of predicted pathways enriched in the same comparisons where many taxa were enriched), so we have focused on the taxonomic differences we identified.

## 3 Discussion

We found large and consistent differences in the microbiome between seagrass and structural mimics both on above‐ and belowground surfaces. This builds on previous work that showed distinction between microbiomes on water and leaf surfaces and sediment and root surfaces (Fahimipour et al. 2017), showing definitively that microbiomes respond not just to plant physical structure but also the biological activity or unique physical substrate associated with the host. Previous work has shown strong geographic variation in the microbiome of seagrasses at medium and large scales (Fahimipour et al. 2017; Hurtado‐McCormick et al. 2019) yet, we find consistent differences between mimics and plants across four geographically‐close sites and three time periods from early to late summer. These findings were consistent for both roots and leaves. Our identification of consistent differences among plants and mimics across time confirms a need to understand how these communities are cultivated/built, their interactions with the plants, and ultimate influence on plant fitness in future efforts.

### 3.1 Leaves Are Differentiated From Passive Mimics

While we saw a > 50% overlap in core taxa between the mimics and leaves, we found that they were compositionally distinct (both taxonomically and functionally) and even showed differences in alpha diversity (more ASVs were found on the average mimic than the average leaf). Epiphytic algae also have higher alpha diversity on mimicked compared to natural seagrass and differences in the relative abundance of major microalgal groups between mimic and leaves (Pinckney and Micheli 1998). These differences in communities between leaves and mimics likely represent either microbial preferences for different surfaces or selection by plants. This could be due to differences in physical texture, differences in inorganic materials due to plant uptake and release, or the presence of exuded organic carbon on the leaf surface. Differences in microalgal epibionts could also affect the surface environment and thus the microbiome. The reduced alpha diversity on real leaves suggests that there may be some selection by the live leaf environment, but also some bacterial preferences as the leaf microbiome is not simply a subset of that on mimics (Figure 1B). While these mimics were not perfect physical mimics, we did find they captured a large portion of the eelgrass leaf community, suggesting a large portion of the eelgrass leaf microbiome are substrate generalists.

Our seagrass mimics contrast with mimicked environments from other marine organismal phyllospheres. Similar work using seagrass leaf mimics in British Columbia has compared core microbial composition of seagrass of different ages to plastic mimics and found communities on mimics resembled older leaves (Sanders‐Smith et al. 2020; Trevizan Segovia et al. 2021). While we found some common taxa between our studies (e.g., *Methylotenera*, *Granulosicoccus*, *Lewinella*), their results indicated that ASVs from these genera were higher on leaf tissue while we found that different ASVs from these genera were abundant on each surface. Notably, our seagrass mimics were placed inside of rather than adjacent to beds and we have a much higher sample count of artificial mimics across multiple sites and times. Additionally, our analyses of differences were based on testing for differences in relative abundance between groups made easier with balanced design and more samples. Recent work in kelp indicated an enrichment of common seawater taxa on artificial substrate (agar infused with and without kelp), no difference in taxonomic diversity on artificial substrates compared to kelp, and increases in aerobic taxa on the surface of kelp blades (Weigel and Pfister 2021). We found none of these patterns on seagrass and seagrass mimics, instead finding higher taxonomic diversity on mimics and no compelling evidence that compositional differences we observed were due to differences in aerobic conditions. The distinction from our study could be driven by the accumulation of epiphytic algal communities on both seagrass leaves and mimics that could render the mimic surfaces highly aerobic; seagrasses release less organic carbon on their surfaces than macroalgae (Barrón et al. 2014) which likely further distinguishes microbe‐host interactions in seagrass from those in kelp. Another seaweed study in *Laminaria* indicates similarity between seaweed and adjacent rock biofilms (Lemay et al. 2021). Our study adds a direct comparison to identify not just overlap in core microbial communities, but direct comparisons of relative abundances of different microbial groups to identify likely targets in future studies and manipulations. We think that using these methods will allow more rigorous comparisons between microbial community makeup, and also suggest close attention to detail of comparison substrate when considering a physical or biological mimics to avoid selecting for that surface versus a more passive substrate (Sanders‐Smith et al. 2020).

For terrestrial plant leaf microbiomes, leaf structural details are critical (Doan and Leveau 2015) because plant leaf morphology influences microbiota via moisture retention (Doan et al. 2020), a mechanism that is irrelevant to the submerged microbiome of aquatic plant leaves. Like leaves of terrestrial plants, seagrass leaves exude amino acids (Jørgensen et al. 1981) and dissolved organic carbon (Wetzel and Penhale 1979) which create a uniquely rich environment potentially shaping their microbial communities including some predicted amino acid degradation pathways we identified (Table S4). Future experiments could target the role of released metabolites in assembling seagrass leaf microbiomes as has been explored in terrestrial systems (Zhang et al. 2014; Doan and Leveau 2015; Warning and Datta 2017) to compare differences that have arisen with the transition to the sea.

Finally, while our limited functional evidence does not indicate clear functional differences, the specific taxa that we observe on leaves may have speculatively important roles that are worthy of further investigation. The repeated enrichment of certain ASVs on leaves versus mimics (especially where they are absent in mimics such as an ASV of *Kordia* or several genera in Saprospiraceae) suggests that they might be good targets for experimentation. Some—such as *Kordia* spp.—are likely algicidal bacteria that have previously been isolated during red tides where they showed algicidal activity to *Skeletonema costatum* and multiple other algal species (Sohn et al. 2004). We have previously identified different *Kordia* ASVs at higher relative abundance under different temperature regimes which might indicate niche differentiation (Schenck et al. 2022). Potentially, *Kordia* species could be cultivated by seagrass to capitalise on their algicidal properties and manage epiphyte loads. The two Saprospiraceae that we saw multiple representatives within a genera only present on leaves (*Lewinella* spp. and *Rubidimonas* spp.) are both genera comprised of aerobic heterotrophs previously isolated from marine environments that can metabolise complex starches (McIlroy and Nielsen 2014) and have previously been associated higher levels in macroalgal diseases (Zozaya‐Valdés et al. 2017). While these bacteria are associated more strongly with seagrass, further investigation is needed to determine how they interact with seagrass, seagrass diseases and seagrass epiphytes—as they may be cultivated partners in removing unwanted epiphytes, could be parasites, or may simply be commensal residents of seagrass or its epiphytes.

### 3.2 Root Microbes Are Vastly Different From Those in Sediment and on Root Mimics

That root microbiomes differed from mimics was not surprising given that roots exude both organic (e.g., sugars) and inorganic (e.g., oxygen) compounds that have major influences on microbiota. In fact, sugar concentrations associated with seagrass roots can be exceedingly high yet may not be metabolised by bacteria due to the presence of inhibitory phenolic compounds (Sogin et al. 2022). Similarly, the combination of oxygen leakage from root tips and surrounding sulphidic sediments promotes sulphur oxidising bacteria (Brodersen et al. 2018; Martin et al. 2019). Given these plant‐caused environmental differences from surrounding sediments, it does not seem surprising that root mimics microbiomes did not resemble those on roots. However, as in leaves, we captured the surface microbiome of a physical structure buried in sediment. There was no clear ‘core’ of root mimic communities and while there was no evidence of a difference in alpha diversity, our taxonomic analyses showed that taxa that varied between mimics and sediments or roots were generally higher in relative abundance on root surfaces or in sediments than on the mimics. Given the lower abundances, we were surprised to see the opposite result in functional predictions where predicted pathways were generally upregulated on mimics. However, given the more limited functional predictions (including the lack of upregulation sulphate reduction related predicted pathways on roots and sediments compared to mimics despite increases in taxonomic relative abundance of sulphate reducing bacteria) and rapidly changing taxonomy of some of these groups (Waite et al. 2020), the bacteria on mimics might be better described than other groups. However, based on taxonomy we have identified several families containing ASVs worthy of further investigation including Desulfobacteraceae and Prolixibacteraceae, both of which were overrepresented on roots compared to mimics and sediments and involved in sulphate‐reduction and nitrogen cycling respectively.

Terrestrial experiments investigating rhizosphere microbial communities through creation of artificial environments have added root exudates through capillaries (e.g., addition of oxalic acid into soils frees carbon; Keiluweit et al. 2015), and similar experiments could test the roles of exudates and oxygen extrusion independently and in combination to further determine the mechanisms by which microbial communities on the surfaces of roots are assembled, particularly how they sustain these plants in highly anoxic sediments. Based on the taxa we identified, it is likely that seagrass root bacterial communities, like those of terrestrial plants, are structured by a resource exchange between hosts and microbes. While there are many differences in what these resources are and partnerships look like (e.g., eelgrass do not have associations with arbuscular mycorrhizal fungi (Nielsen et al. 1999; Ettinger and Eisen 2019) and land plants live in well oxygenated soils instead of sulphidic anoxic sediments), aquatic and land angiosperms participate in a resource exchange that drives microbial community structures in the rhizosphere (for review of land plants, see Jacoby et al. 2017). This suggests that it is likely that root microbes are not only affected by the eelgrass, but affect eelgrass itself, perhaps through sulphur metabolism (Fahimipour et al. 2017). While no studies have directly tested the effect of root microbiome on seagrass growth, the oxidation of sulphides by lucinid bivalve—bacterial symbiosis has been implicated as a major influence on seagrass success in anoxic sediments (van der Heide et al. 2012). Direct tests implicating changing rhizosphere microbial communities with changes in plant performance would be necessary to explicitly test these roles but starting with isolates from many of the families we saw preferentially on seagrass roots compared to mimics and sediments seem likely candidates for future experimental tests.

### 3.3 Overall Conclusions

We found robust evidence of differences between mimics and plant tissues in seagrass. These large differences suggest that there is a unique environment created by the plant that creates these distinct communities either as part of active partnerships or through inhibition of certain microbes or unique characteristics of that environment preferred by some microbes. Unlike in terrestrial plant surfaces, water retention is unlikely to play a role in driving microbe assembly in aquatic leaves. Seagrass leaf microbiomes may be structured more like microbiomes in terrestrial and marine root microbiomes, by exudates of plants and their influence on the environment. Further experiments with more detailed or realistic mimics could isolate the mechanisms by which these microbiomes are structured and function.

Finally, despite the differences emphasised here, there is vast overlap between these communities even on simple substrates, especially on leaves and their mimics. This designates a manageable number of taxa to further examine to see what factors are driving their unique assemblies. With a continued and rising interest in microbial communities in general and the role of microbial communities associated with seagrasses specifically, extensions of this work point towards associations that should be further explored for understanding host–microbiome dynamics across species ranges.

## 4 Experimental Procedures

### 4.1 Field Methods

In July 2015, we deployed artificial substrates to characterise the microbiome of leaf and root mimics compared with those on live plants (see Figure S3 for site locations). We conducted this experiment at four eelgrass beds within Bodega Harbor with increasing distance from the mouth of the harbour, along a gradient of increasing temperature (2°C mean temperature difference among sites), decreasing water flow, and progressively finer sediment grain size that result in each site harbouring distinct eelgrass microbial communities on seagrass that vary among seasons (Kardish and Stachowicz 2023). We used 0.75 m long, 4 mm wide green polypropylene ribbons attached to a vexar mesh anchored into sediments to mimic artificial leaf substrates, a standard technique that has been used for over 40 years to mimic the physical habitat provided by eelgrass to isolate the role of physical structure in structuring the epiphytic, epifaunal and fish communities that inhabit eelgrass (Barber et al. 1979). These ribbons mimic the physical structure of seagrass leaves in a bed with a similar length, width and accumulation of epiphytes. We also deployed artificial root substrates (10 cm twist ties twisted in half around the same vexar mesh as ribbons) inserted into the sediment to the depth where most root biomass is found at these sites (approximately 2–5 cm deep) to examine the community that accumulates on a physical structure at a similar depth in sediment. Neither artificial substrate was preinoculated with microbial communities, but they were deployed inside a seagrass bed immediately adjacent to live plants. We sampled undisturbed plants near the mimics for comparison. We sampled one, 2 and 3 months after deployment, which is sufficient time for transplanted eelgrass to take on microbial characteristics of a new site (Kardish and Stachowicz 2023). For leaves and leaf mimics, we took a 2 cm clip of leaf or ribbon at approximately 15 cm above the sediment surface, which is approximately the same height as where leaf samples were taken. For the root mimics, we took a 2 cm clip from the bottom of the twist tie (at approximately the same depth as root samples). For roots, we detached ~ 10 roots from the rhizome. For sediment samples, we took a small sediment sample from approximately 2 cm under the sediment surface (a similar depth to roots sampled). We immediately placed samples on dry ice and froze them at −80°C within a few hours of sampling until extraction, and all instruments were sterilised with 70% isopropyl alcohol before taking samples. At each of the four sites, across three each of three timepoints, we sampled three sediment samples, three mimic samples (for each of leaf and root mimics), and four plant samples (leaf and root). Due to the identifiable nature of these sample types, we were unable to blind ourselves to sample type during sampling or extraction.

To confirm that the differences we saw were not due to the introduction of mimics at a site attached to these screens, we repeated this analysis with disturbed plants that were taken from these sites, taken to Bodega Marine Lab, attached to the same vexar screens as the mimics and planted back into the field. These results parallel the results presented in the main text comparing mimics and undisturbed plants; parallel analyses can be found in Appendix S1.

### 4.2 Molecular Methods and Bioinformatic Analysis

We extracted DNA with the MoBio PowerSoil DNA kit from leaves, roots, and sediments. To get the surface of the leaves and roots only, we vortexed each frozen sample with 500ul of MilliQ water and then added that liquid to the bead tubes and proceeded with the standard extraction protocol (full protocol available at github.mkardish/Transplants/Lab_Protocols). For sediments, we added a small amount of sediment (approximately 0.25 mg) directly to the bead tube. We amplified and sequenced the V4‐V5 region of the 16S rRNA gene on an Illumina MiSeq to identify bacteria present at the Integrated Microbiome Resource at Dalhousie University with primers 515F and 926R (Walters et al. 2016; Comeau et al. 2017).

### 4.3 Bioinformatic Analysis

We ran all bioinformatic and statistical analyses in R (version 4.0.3). We used a standard dada2 pipeline to error check our reads and to identify amplicon sequence variants (Callahan et al. 2016). We used only forward reads in our subsequent analyses (280 base pairs). We identified ASV taxonomy based on the SILVA database (Quast et al. 2013) and built a phylogeny of ASVs using alignments built with DECIPHER (Wright 2015) then a tree built with FastTree2 (Price et al. 2010) then converted to ultrametric (Britton et al. 2007). We then rooted the bacterial tree with an archaeal outgroup (Callahan et al. 2016). We identified core ASVs as those present in at least 50% of samples of a type with at least 1% detection rate, this means that these ASVs are present in the majority of samples of a given type with some level abundance. We aimed to detect abundant members of a diverse community when defining the core.

We also examined the functional potential of the metagenomes of our samples using PICRUSt2 (Douglas et al. 2020). While these predictions come with major caveats for environmental samples due to underrepresentation in the database, we used this approach to infer potential metabolic pathways based on similarities to known metabolisms and compare these among tissue types.

### 4.4 Sampling and Sequencing Success

We identified 7696 bacterial ASVs across 192 leaf, root, mimic and sediment samples after quality filtering samples to 3,752,142 reads. Root samples contained between 390 and 1013 bacterial ASVs on their surface (we measured 47 root samples with read depth between 11,522 and 49,735 reads), Root mimics samples contained between 81 and 790 bacterial ASVs on their surface (we measured 26 root mimic samples with read depth between 2037 and 37,793 reads), Sediment samples contained between 270 and 843 bacterial ASVs on their surface (we measured 36 sediment root samples with read depth between 10,771 and 40,343 reads), leaf samples contained between 191 and 841 bacterial ASVs on their surface (we measured 48 leaf samples with read depth between 5961 and 61,896 reads) and leaf mimic samples which contained between 195 and 717 bacterial ASVs (35 leaf mimic samples with between 4587 and 31,648 reads per sample).

### 4.5 Statistical Analyses

We analysed the compositional changes in our dataset based on phylogenetic similarity among samples by normalising samples via a phylogenetic isometric log transform described in (Silverman et al. 2017) and implemented in the R‐package ‘philr’. This allows a compositional transformation of the phylogenetic data—comparing differential weights at nodes throughout the bacterial tree as opposed to just ASVs. We then calculated the Euclidean distance among samples before using PERMANOVA (via the *adonis2* function in ‘vegan’; Oksanen et al. 2025) to determine differences among sample types controlling for month and site by constraining permutations. We tested homogeneity of group dispersions with the betadispr function in ‘vegan’.

To measure bacterial richness, we rarified all samples to 2950 reads samples which we repeated 200 times (McMurdie and Holmes 2014) and used each sample's average ‘Observed ASVs’ in our analysis as our measure of bacterial richness in a sample. We tested differences in Observed ASVs using the negative binomial mixed model with crossed random effects implemented in lme4: Observed ASVs ~ Sample Type + (Sample Type | Site) + (Sample Type | Month) (Bates et al. 2015). We also visualised overlap in these observed ASVs using upSet which allowed us to identify the numbers of overlapping and non‐overlapping ASVs across sample types (Conway et al. 2017).

To identify which ASVs varied between samples we performed a likelihood ratio test in DESeq2 comparing models of ~ Site + Month + Sample Type with ~ Site + Month (separately for above and belowground samples) after geometric mean centring raw ASV abundances using an alpha of 0.05 and an log‐fold‐change threshold of 0.5 (Love et al. 2014). We then examined the contrast between sample types to identify which ASVs varied in each compartment.

We treated functional data compositionally as well, using PERMANOVA to analyse differences in pathway composition among samples after a centred log‐ratio transformation. We then used DESeq2 with the same models as for taxonomic differences to test for predicted pathway differences among sample types.

## Author Contributions

**Melissa R. Kardish:** conceptualization, visualization, writing – original draft, writing – review and editing, methodology, formal analysis, investigation. **John J. Stachowicz:** writing – review and editing, conceptualization, methodology, supervision, investigation.

## Conflicts of Interest

The authors declare no conflicts of interest.

## Supporting information


Appendix S1.

Data S1.

**Table S7.** For belowground bacterial communities, all ASVs that varied significantly among mimics, seagrass and sediment determined by DESeq2, including magnitude of differences.

## Data Availability

All scripts used to analyse this data are available at www.github.com/mkardish/Mimics and sequences have been deposited under the NCBI BioProject ID PRJNA731931.
