# Supplementary material for: More Than a Stick in the Mud: Eelgrass Leaf and Root Bacterial Communities Are Distinct From Those on Physical Mimics
Source: Environ Microbiol Rep. 2025 Apr 30;17(3):e70086. doi: 10.1111/1758-2229.70086 (PMC12042217; doi:10.1111/1758-2229.70086)
Supplement: Supplementary file 2 — Data S1. [file EMI4-17-e70086-s002.docx]

Supplemental Figures and Tables

**Figure S1**: (A) Mean amplicon sequence variant (ASV) richness found in each type of sample we measured split by timepoint and site sampled (indicated on x-axis). Colors indicate sample type – bright green are leaf mimics, dark green are leaves, red-orange points are root mimics, dark brown points are roots, and dark grey points are sediments, Raw data as well as means and standard errors are presented. (B) Ordination of bacterial community structure based on principal coordinate analysis of phylogenetic-isometric log-ratio transformed distances. Ellipses show leaf and mimic differences observed in Figure 2A. Sites and timepoints are denoted by shape and color respectively. (C) Ordination of bacterial community structure based on principal coordinate analysis of phylogenetic-isometric log-ratio transformed distances. Ellipses show root, mimic, and sediment differences observed in Figure 3A. Sites and timepoints are denoted by shape and color respectively. Timepoints are Month 1 (July), Month 2 (August), Month 3 (September). Sites are CC (Campbell Cove), DB (Doran Beach), MM (Mason’s Marina), and WP (Westside Park).

**
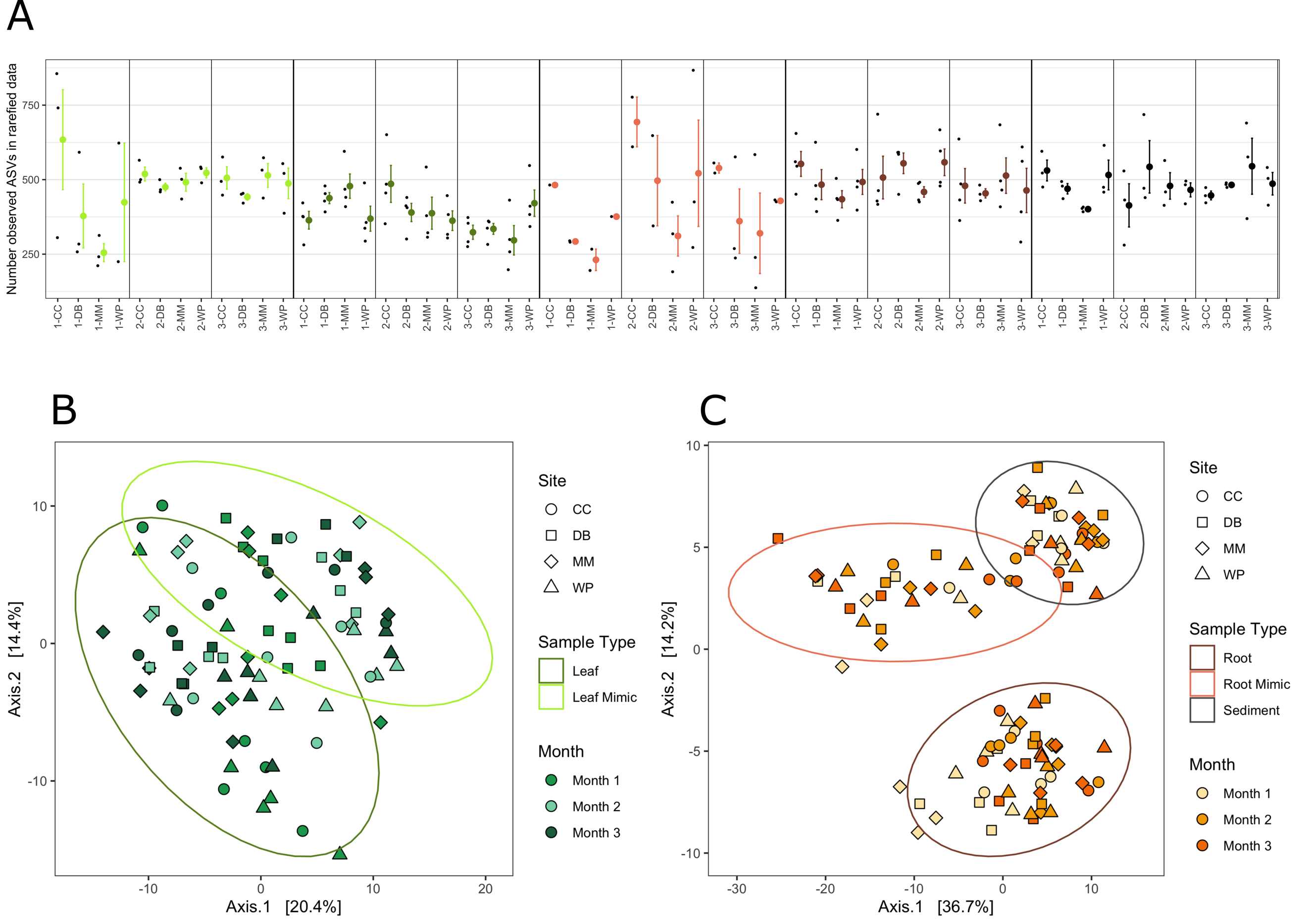
**

**Figure S2**: Overlap among all ASVs present in each sample type. Diagram is a barplot of shared community memberships, equivalent to a Venn diagram.


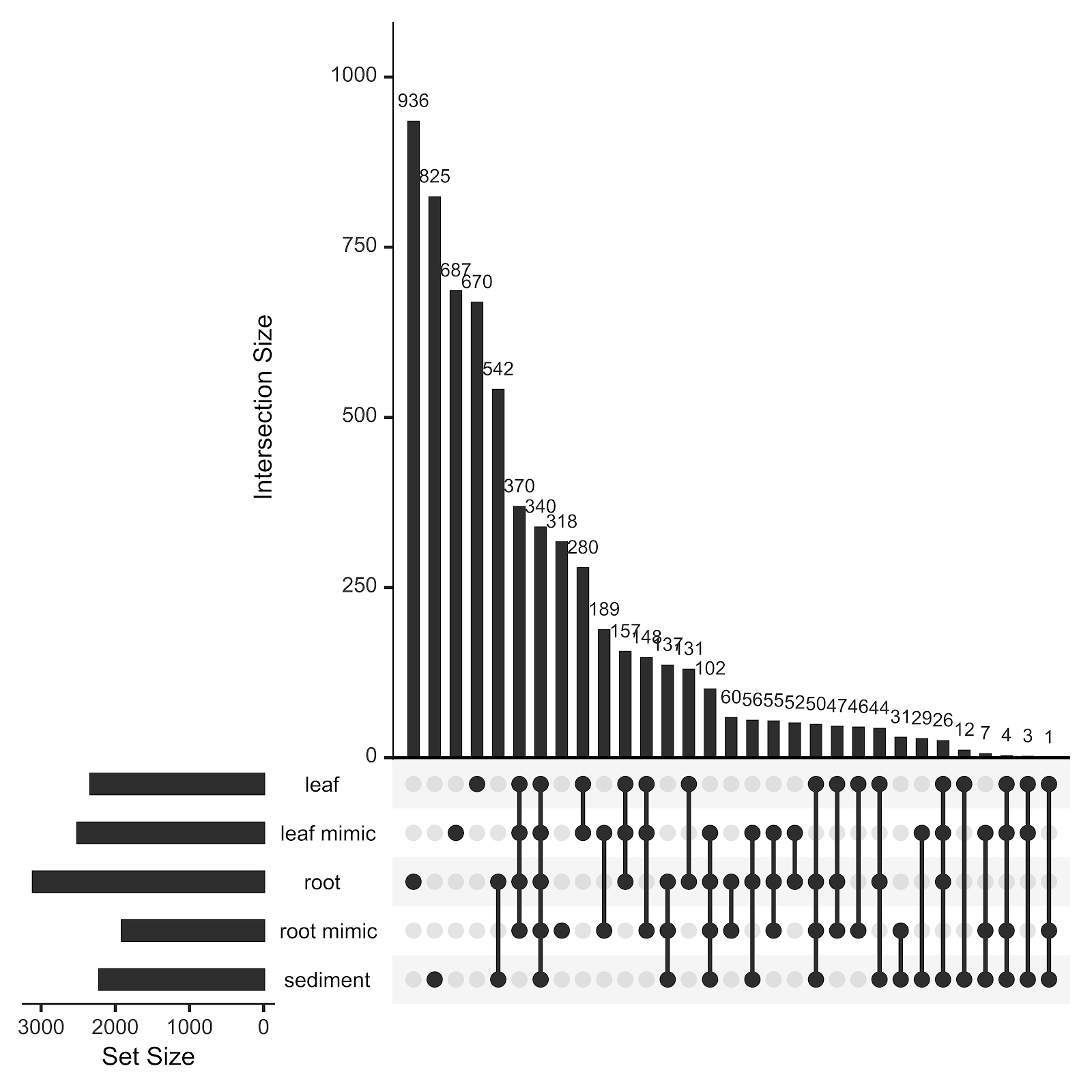


Figure S3: Map of Sampling sites in Bodega Harbor, Bodega Bay, CA, USA.


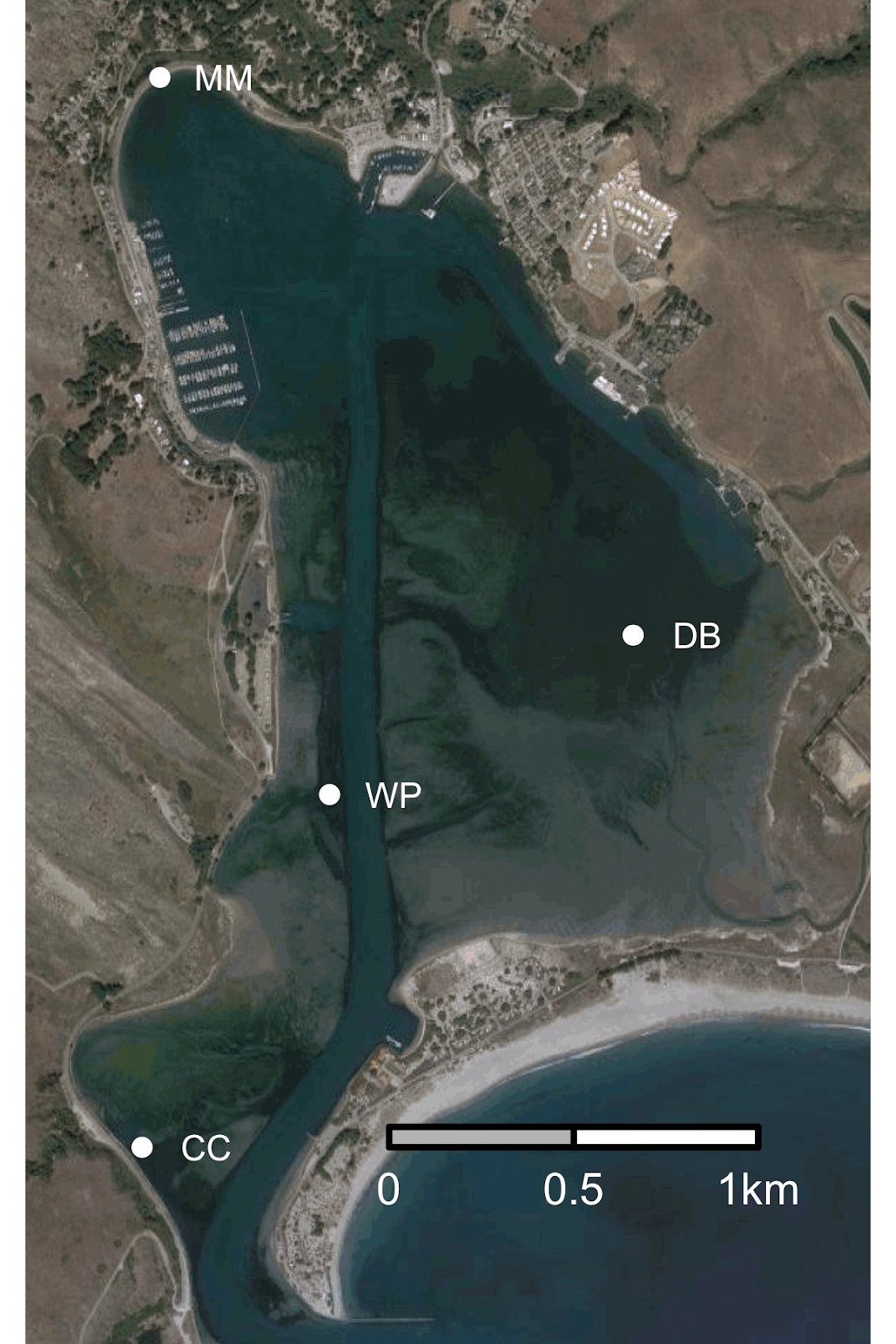


**Supplemental Tables legends (followed by supplemental tables themselves)**

Table S1: For leaf bacterial communities, the family-level identification of ASVs that varied significantly between mimics and seagrass substrate determined by DESeq2.

Table S2:  For leaf bacterial communities, the genus-level identification of ASVs that varied significantly between mimics and seagrass substrate determined by DESeq2.

Table S3: For leaf bacterial communities, all ASVs that varied significantly between mimics and seagrass substrate determined by DESeq2, including magnitude of differences.

Table S4:  For leaf bacterial communities, all Metacyc predicted pathways that varied significantly between mimics and seagrass substrate determined by DESeq2, including magnitude of differences.

Table S5: Results of pairwise PERMANOVA tests distinguishing compositional differences among roots, root mimics, and sediments in both ASV composition and composition of predicted Metacyc pathways.

Table S6: For belowground bacterial communities, the family-level identification of ASVs that varied significantly among mimics, seagrass and sediment determined by DESeq2.

Table S7: For belowground bacterial communities, all ASVs that varied significantly among mimics, seagrass and sediment determined by DESeq2, including magnitude of differences.

Table S8:  For belowground bacterial communities, all Metacyc predicted pathways that varied significantly among mimics, seagrass and sediment determined by DESeq2, including magnitude of differences.

**Tables:**

**Table S1:**

| **Family** | **Higher on leaves** | **Higher on mimics** |
| --- | --- | --- |
| 37-13 | 1 | 0 |
| Alteromonadaceae | 6 | 0 |
| Arenicellaceae | 1 | 2 |
| Bdellovibrionaceae | 1 | 0 |
| Blastocatellaceae | 0 | 1 |
| Caldilineaceae | 1 | 0 |
| Cellvibrionaceae | 1 | 0 |
| Chromatiaceae | 0 | 1 |
| Colwelliaceae | 3 | 0 |
| Crocinitomicaceae | 6 | 0 |
| Cryomorphaceae | 7 | 0 |
| Desulfobulbaceae | 0 | 1 |
| Desulfocapsaceae | 1 | 5 |
| Desulfosarcinaceae | 0 | 1 |
| DEV007 | 0 | 4 |
| Flavobacteriaceae | 10 | 17 |
| Fokiniaceae | 1 | 0 |
| Gimesiaceae | 0 | 1 |
| Granulosicoccaceae | 4 | 2 |
| Halieaceae | 0 | 1 |
| Hyphomicrobiaceae | 0 | 1 |
| Hyphomonadaceae | 3 | 2 |
| Ilumatobacteraceae | 0 | 1 |
| Kangiellaceae | 1 | 0 |
| Legionellaceae | 0 | 1 |
| Magnetospiraceae | 1 | 0 |
| Marinomonadaceae | 1 | 0 |
| Methylophagaceae | 1 | 0 |
| Methylophilaceae | 6 | 1 |
| Micavibrionaceae | 1 | 2 |
| Microtrichaceae | 2 | 3 |
| Nitrincolaceae | 2 | 0 |
| Nitrosococcaceae | 1 | 0 |
| NS11-12_marine_group | 1 | 0 |
| NS9_marine_group | 1 | 0 |
| Oleiphilaceae | 1 | 0 |
| Phormidesmiaceae | 0 | 1 |
| Pirellulaceae | 7 | 2 |
| Pseudohongiellaceae | 1 | 0 |
| Rhizobiaceae | 1 | 7 |
| Rhizobiales_Incertae_Sedis | 0 | 1 |
| Rhodobacteraceae | 31 | 20 |
| Rhodothermaceae | 1 | 0 |
| Rickettsiaceae | 1 | 0 |
| Rubinisphaeraceae | 1 | 3 |
| Rubritaleaceae | 0 | 4 |
| Saprospiraceae | 26 | 1 |
| Schleiferiaceae | 1 | 0 |
| Shewanellaceae | 0 | 1 |
| Sphingomonadaceae | 0 | 2 |
| Spirosomaceae | 1 | 0 |
| Spongiibacteraceae | 1 | 0 |
| Sulfurimonadaceae | 1 | 0 |
| Sulfurovaceae | 0 | 4 |
| Terasakiellaceae | 1 | 0 |
| Thiomicrospiraceae | 1 | 0 |
| Thiotrichaceae | 0 | 1 |
| Trueperaceae | 0 | 1 |
| Unknown_Family | 0 | 2 |
| Woeseiaceae | 0 | 4 |

**Table S2**

| **Family** | **Genus** | **Higher on leaves** | **Higher on mimics** |
| --- | --- | --- | --- |
| Alteromonadaceae | Glaciecola | 5 | 0 |
| Alteromonadaceae | Salinimonas | 1 | 0 |
| Arenicellaceae | Arenicella | 1 | 2 |
| Bdellovibrionaceae | OM27_clade | 1 | 0 |
| Blastocatellaceae | Blastocatella | 0 | 1 |
| Cellvibrionaceae | Agaribacterium | 1 | 0 |
| Chromatiaceae | Halochromatium | 0 | 1 |
| Colwelliaceae | Colwellia | 3 | 0 |
| Crocinitomicaceae | Crocinitomix | 1 | 0 |
| Crocinitomicaceae | Fluviicola | 3 | 0 |
| Cryomorphaceae | NS10_marine_group | 1 | 0 |
| Cryomorphaceae | Vicingus | 2 | 0 |
| Desulfobulbaceae | Desulfobulbus | 0 | 1 |
| Desulfosarcinaceae | Sva0081_sediment_group | 0 | 1 |
| Flavobacteriaceae | Aurantivirga | 1 | 0 |
| Flavobacteriaceae | Changchengzhania | 1 | 0 |
| Flavobacteriaceae | Dokdonia | 1 | 0 |
| Flavobacteriaceae | Kordia | 3 | 0 |
| Flavobacteriaceae | NS3a_marine_group | 1 | 0 |
| Flavobacteriaceae | Polaribacter | 1 | 0 |
| Flavobacteriaceae | Ulvibacter | 2 | 3 |
| Flavobacteriaceae | Actibacter | 0 | 1 |
| Flavobacteriaceae | Aquibacter | 0 | 2 |
| Flavobacteriaceae | Jejudonia | 0 | 1 |
| Flavobacteriaceae | Maribacter | 0 | 2 |
| Flavobacteriaceae | Psychroserpens | 0 | 1 |
| Flavobacteriaceae | Robiginitalea | 0 | 1 |
| Flavobacteriaceae | Winogradskyella | 0 | 2 |
| Fokiniaceae | MD3-55 | 1 | 0 |
| Granulosicoccaceae | Granulosicoccus | 4 | 2 |
| Halieaceae | Halioglobus | 0 | 1 |
| Hyphomicrobiaceae | Filomicrobium | 0 | 1 |
| Hyphomonadaceae | Hellea | 1 | 0 |
| Hyphomonadaceae | Litorimonas | 1 | 0 |
| Hyphomonadaceae | Robiginitomaculum | 1 | 0 |
| Hyphomonadaceae | Hyphomonas | 0 | 1 |
| Ilumatobacteraceae | Ilumatobacter | 0 | 1 |
| Marinomonadaceae | Marinomonas | 1 | 0 |
| Methylophilaceae | Methylotenera | 6 | 1 |
| Microtrichaceae | Sva0996_marine_group | 1 | 3 |
| Nitrosococcaceae | Cm1-21 | 1 | 0 |
| Oleiphilaceae | Oleiphilus | 1 | 0 |
| Phormidesmiaceae | Phormidesmis_ANT.LACV5.1 | 0 | 1 |
| Pirellulaceae | Blastopirellula | 4 | 1 |
| Pirellulaceae | Rhodopirellula | 2 | 1 |
| Pirellulaceae | Rubripirellula | 1 | 0 |
| Pseudohongiellaceae | Pseudohongiella | 1 | 0 |
| Rhizobiaceae | Pseudahrensia | 1 | 3 |
| Rhizobiaceae | Ahrensia | 0 | 1 |
| Rhizobiaceae | Hoeflea | 0 | 1 |
| Rhizobiales_Incertae_Sedis | Anderseniella | 0 | 1 |
| Rhodobacteraceae | Aliiroseovarius | 1 | 0 |
| Rhodobacteraceae | Octadecabacter | 1 | 1 |
| Rhodobacteraceae | Pacificibacter | 1 | 0 |
| Rhodobacteraceae | Planktotalea | 1 | 0 |
| Rhodobacteraceae | Sedimentitalea | 1 | 1 |
| Rhodobacteraceae | Sulfitobacter | 2 | 2 |
| Rhodobacteraceae | Tateyamaria | 1 | 1 |
| Rhodobacteraceae | Yoonia-Loktanella | 4 | 0 |
| Rhodobacteraceae | Celeribacter | 0 | 1 |
| Rhodobacteraceae | Jannaschia | 0 | 1 |
| Rhodobacteraceae | Leisingera | 0 | 1 |
| Rhodobacteraceae | Litoreibacter | 0 | 1 |
| Rhodobacteraceae | Phaeobacter | 0 | 1 |
| Rhodobacteraceae | Roseobacter | 0 | 1 |
| Rhodobacteraceae | Roseovarius | 0 | 1 |
| Rhodobacteraceae | Thiobacimonas | 0 | 1 |
| Rhodobacteraceae | Tropicimonas | 0 | 1 |
| Rickettsiaceae | Candidatus_Megaira | 1 | 0 |
| Rubinisphaeraceae | Fuerstia | 1 | 0 |
| Rubinisphaeraceae | Planctomicrobium | 0 | 2 |
| Rubritaleaceae | Haloferula | 0 | 1 |
| Rubritaleaceae | Persicirhabdus | 0 | 2 |
| Rubritaleaceae | Roseibacillus | 0 | 1 |
| Saprospiraceae | Lewinella | 3 | 0 |
| Saprospiraceae | Phaeodactylibacter | 1 | 0 |
| Saprospiraceae | Portibacter | 2 | 0 |
| Saprospiraceae | Rubidimonas | 3 | 0 |
| Schleiferiaceae | Schleiferia | 1 | 0 |
| Shewanellaceae | Shewanella | 0 | 1 |
| Sphingomonadaceae | Parasphingopyxis | 0 | 1 |
| Spirosomaceae | Taeseokella | 1 | 0 |
| Sulfurimonadaceae | Sulfurimonas | 1 | 0 |
| Sulfurovaceae | Sulfurovum | 0 | 4 |
| Thiomicrospiraceae | endosymbionts | 1 | 0 |
| Thiotrichaceae | Cocleimonas | 0 | 1 |
| Trueperaceae | Truepera | 0 | 1 |
| Unknown_Family | Marinicella | 0 | 1 |
| Woeseiaceae | Woeseia | 0 | 4 |

**Table S3**

| **Family** | **Genus** | **Species** | **Base**  **Mean** | **log2**  **Fold**  **Change** | **lfcSE** | **stat** | **pvalue** | **padj** |
| --- | --- | --- | --- | --- | --- | --- | --- | --- |
| Blastocatellaceae | Blastocatella | NA | 8.634 | -3.969 | 0.953 | 13.066 | 0 | 0.003 |
| Pirellulaceae | Rhodopirellula | NA | 4.417 | -3.264 | 1.125 | 7.653 | 0.006 | 0.03 |
| Pirellulaceae | Rubripirellula | NA | 8.841 | 7.502 | 1.265 | 6.431 | 0.011 | 0.05 |
| Pirellulaceae | Rhodopirellula | NA | 12.059 | 3.583 | 0.763 | 17.452 | 0 | 0 |
| Pirellulaceae | Blastopirellula | NA | 26.01 | 2.218 | 0.453 | 16.77 | 0 | 0.001 |
| Pirellulaceae | Rhodopirellula | NA | 11.134 | 1.897 | 0.549 | 9.302 | 0.002 | 0.015 |
| Pirellulaceae | Blastopirellula | NA | 6.362 | -3.07 | 0.656 | 16.339 | 0 | 0.001 |
| Pirellulaceae | Blastopirellula | NA | 21.227 | 2.555 | 0.594 | 14.095 | 0 | 0.002 |
| Phormidesmiaceae | Phormidesmis_ANT.LACV5.1 | NA | 58.235 | -2.408 | 0.557 | 12.483 | 0 | 0.003 |
| Rubinisphaeraceae | Planctomicrobium | NA | 8.024 | -4.426 | 0.76 | 21.124 | 0 | 0 |
| Rubinisphaeraceae | NA | NA | 29.24 | -1.711 | 0.341 | 21.782 | 0 | 0 |
| Rubinisphaeraceae | Planctomicrobium | NA | 7.738 | -3.777 | 0.895 | 11.251 | 0.001 | 0.006 |
| Rubinisphaeraceae | Fuerstia | NA | 11.297 | 2.542 | 0.808 | 7.228 | 0.007 | 0.036 |
| Gimesiaceae | NA | NA | 8.264 | -4.048 | 0.964 | 11.668 | 0.001 | 0.005 |
| Pirellulaceae | Blastopirellula | NA | 36.821 | 8.175 | 0.608 | 116.911 | 0 | 0 |
| Pirellulaceae | Blastopirellula | NA | 11.206 | 4.985 | 0.892 | 10.461 | 0.001 | 0.009 |
| Sulfurimonadaceae | Sulfurimonas | NA | 7.24 | 2.283 | 0.779 | 7.123 | 0.008 | 0.037 |
| Sulfurovaceae | Sulfurovum | NA | 35.1 | -2.351 | 0.432 | 26.554 | 0 | 0 |
| Sulfurovaceae | Sulfurovum | NA | 4.178 | -3.675 | 1.385 | 6.476 | 0.011 | 0.049 |
| Sulfurovaceae | Sulfurovum | NA | 11.242 | -3.427 | 1.053 | 8.87 | 0.003 | 0.018 |
| Sulfurovaceae | Sulfurovum | NA | 25.418 | -2.611 | 0.594 | 17.23 | 0 | 0 |
| Rubritaleaceae | Persicirhabdus | NA | 17.136 | -3.59 | 0.674 | 16.305 | 0 | 0.001 |
| Rubritaleaceae | Persicirhabdus | NA | 3.481 | -4.442 | 1.483 | 7.128 | 0.008 | 0.037 |
| Rubritaleaceae | Haloferula | NA | 8.991 | -2.561 | 0.758 | 6.959 | 0.008 | 0.04 |
| DEV007 | NA | NA | 2.813 | -4.186 | 1.106 | 12.838 | 0 | 0.003 |
| DEV007 | NA | NA | 4.844 | -3.843 | 1.037 | 9.461 | 0.002 | 0.014 |
| DEV007 | NA | NA | 2.053 | -3.383 | 1.084 | 7.955 | 0.005 | 0.026 |
| DEV007 | NA | NA | 10.793 | -4.078 | 0.752 | 15.57 | 0 | 0.001 |
| Granulosicoccaceae | Granulosicoccus | NA | 28.28 | 7.429 | 0.607 | 97.66 | 0 | 0 |
| Granulosicoccaceae | Granulosicoccus | coccoides | 58.104 | 2.262 | 0.45 | 17.754 | 0 | 0 |
| Granulosicoccaceae | Granulosicoccus | NA | 22.483 | 4.112 | 0.789 | 18.559 | 0 | 0 |
| Granulosicoccaceae | Granulosicoccus | NA | 105.233 | -1.658 | 0.342 | 17.461 | 0 | 0 |
| Granulosicoccaceae | Granulosicoccus | NA | 119.671 | 2.337 | 0.413 | 22.214 | 0 | 0 |
| Granulosicoccaceae | Granulosicoccus | NA | 14.532 | -4.847 | 1.192 | 15.868 | 0 | 0.001 |
| Arenicellaceae | Arenicella | NA | 41.303 | 6.26 | 0.618 | 62.286 | 0 | 0 |
| Arenicellaceae | Arenicella | NA | 3.611 | -4.538 | 1.567 | 6.967 | 0.008 | 0.04 |
| Arenicellaceae | Arenicella | NA | 9.769 | -4.871 | 1.062 | 17.243 | 0 | 0 |
| Nitrosococcaceae | Cm1-21 | NA | 6.562 | 2.399 | 0.723 | 7.883 | 0.005 | 0.027 |
| Woeseiaceae | Woeseia | NA | 6.946 | -5.372 | 1.229 | 11.785 | 0.001 | 0.005 |
| Woeseiaceae | Woeseia | NA | 9.432 | -3.913 | 0.858 | 8.532 | 0.003 | 0.02 |
| Woeseiaceae | Woeseia | NA | 14.759 | -2.914 | 0.875 | 10.289 | 0.001 | 0.009 |
| Woeseiaceae | Woeseia | NA | 16.331 | -2.514 | 0.735 | 6.701 | 0.01 | 0.045 |
| Chromatiaceae | Halochromatium | NA | 13.673 | -2.491 | 0.71 | 10.648 | 0.001 | 0.008 |
| Spongiibacteraceae | NA | NA | 58.424 | 3.862 | 0.537 | 34.029 | 0 | 0 |
| Unknown_Family | NA | NA | 53.935 | -1.182 | 0.296 | 13.938 | 0 | 0.002 |
| Halieaceae | Halioglobus | NA | 21.326 | -3.818 | 0.856 | 7.886 | 0.005 | 0.027 |
| Pseudohongiellaceae | Pseudohongiella | NA | 4.611 | 3.956 | 1.297 | 7.377 | 0.007 | 0.033 |
| Methylophilaceae | Methylotenera | NA | 19.786 | -3.236 | 0.849 | 13.234 | 0 | 0.002 |
| Methylophilaceae | Methylotenera | NA | 61.362 | 5.868 | 0.586 | 62.869 | 0 | 0 |
| Methylophilaceae | Methylotenera | NA | 13.359 | 5.89 | 1.13 | 16.955 | 0 | 0 |
| Methylophilaceae | Methylotenera | NA | 10.382 | 6.331 | 1.785 | 8.445 | 0.004 | 0.021 |
| Methylophilaceae | Methylotenera | NA | 11.383 | 7.854 | 1.057 | 7.915 | 0.005 | 0.027 |
| Methylophilaceae | Methylotenera | NA | 63.597 | 1.297 | 0.38 | 9.628 | 0.002 | 0.013 |
| Methylophilaceae | Methylotenera | NA | 83.462 | 5.768 | 0.62 | 50.412 | 0 | 0 |
| Alteromonadaceae | Glaciecola | NA | 5.984 | 3.123 | 1.063 | 7.198 | 0.007 | 0.036 |
| Alteromonadaceae | Salinimonas | NA | 13.51 | 4.726 | 1.031 | 12.954 | 0 | 0.003 |
| Alteromonadaceae | Glaciecola | NA | 11.982 | 5.014 | 1.081 | 15.418 | 0 | 0.001 |
| Alteromonadaceae | Glaciecola | NA | 17.041 | 4.317 | 0.903 | 11.855 | 0.001 | 0.004 |
| Alteromonadaceae | Glaciecola | NA | 37.762 | 6.247 | 0.629 | 61.608 | 0 | 0 |
| Alteromonadaceae | Glaciecola | punicea | 13.312 | 4.5 | 0.919 | 12.536 | 0 | 0.003 |
| Methylophagaceae | NA | NA | 8.874 | 5.272 | 1.02 | 19.437 | 0 | 0 |
| Colwelliaceae | Colwellia | polaris | 58.733 | 6.264 | 0.683 | 45.932 | 0 | 0 |
| Colwelliaceae | Colwellia | NA | 38.304 | 6.45 | 0.695 | 53.797 | 0 | 0 |
| Colwelliaceae | Colwellia | NA | 12.159 | 25.489 | 1.8 | 6.893 | 0.009 | 0.041 |
| Kangiellaceae | NA | NA | 22.289 | 4.652 | 0.662 | 27.473 | 0 | 0 |
| Marinomonadaceae | Marinomonas | NA | 15.231 | 5.285 | 0.987 | 16.701 | 0 | 0.001 |
| Nitrincolaceae | NA | NA | 17.265 | 7.078 | 1.149 | 24.056 | 0 | 0 |
| Nitrincolaceae | NA | NA | 8.395 | 4.688 | 1.013 | 14.109 | 0 | 0.002 |
| Thiotrichaceae | Cocleimonas | NA | 9.583 | -4.236 | 0.86 | 6.445 | 0.011 | 0.05 |
| Shewanellaceae | Shewanella | NA | 8.242 | -2.413 | 0.703 | 8.24 | 0.004 | 0.023 |
| Oleiphilaceae | Oleiphilus | NA | 16.753 | 7.231 | 0.661 | 83.582 | 0 | 0 |
| Cellvibrionaceae | Agaribacterium | NA | 11.934 | 4.209 | 0.84 | 17.029 | 0 | 0 |
| Legionellaceae | NA | NA | 2.351 | -3.483 | 1.241 | 7.071 | 0.008 | 0.038 |
| Unknown_Family | Marinicella | NA | 50.924 | -1.839 | 0.524 | 9.927 | 0.002 | 0.011 |
| Thiomicrospiraceae | endosymbionts | NA | 5.656 | 5.261 | 1.24 | 13.717 | 0 | 0.002 |
| Microtrichaceae | Sva0996_marine_group | NA | 2.904 | -3.319 | 1.132 | 7.58 | 0.006 | 0.031 |
| Trueperaceae | Truepera | NA | 9.138 | -2.689 | 0.554 | 17.499 | 0 | 0 |
| Flavobacteriaceae | Actibacter | NA | 33.825 | -1.63 | 0.463 | 9.545 | 0.002 | 0.013 |
| Flavobacteriaceae | NA | NA | 20.98 | -5.507 | 0.911 | 16.74 | 0 | 0.001 |
| Flavobacteriaceae | Psychroserpens | damuponensis | 159.42 | -1.161 | 0.294 | 14.13 | 0 | 0.002 |
| Flavobacteriaceae | NA | NA | 84.944 | -1.752 | 0.411 | 16.497 | 0 | 0.001 |
| Flavobacteriaceae | Aquibacter | NA | 7.297 | -8.819 | 1.127 | 8.076 | 0.004 | 0.025 |
| Flavobacteriaceae | Aquibacter | NA | 136.29 | -1.432 | 0.311 | 19.408 | 0 | 0 |
| Flavobacteriaceae | Winogradskyella | NA | 26.714 | -6.965 | 1.073 | 9.694 | 0.002 | 0.012 |
| Flavobacteriaceae | Winogradskyella | NA | 52.784 | -3.806 | 0.96 | 6.546 | 0.011 | 0.048 |
| Flavobacteriaceae | Dokdonia | NA | 21.086 | 3.523 | 1.175 | 7.398 | 0.007 | 0.033 |
| Flavobacteriaceae | Ulvibacter | NA | 38.294 | 4.716 | 0.778 | 28.698 | 0 | 0 |
| Flavobacteriaceae | Ulvibacter | NA | 185.979 | -1.902 | 0.48 | 9.204 | 0.002 | 0.015 |
| Flavobacteriaceae | Changchengzhania | NA | 56.506 | 2.425 | 0.504 | 15.731 | 0 | 0.001 |
| Flavobacteriaceae | Ulvibacter | NA | 41.038 | 5.34 | 0.749 | 22.334 | 0 | 0 |
| Flavobacteriaceae | Ulvibacter | NA | 32.722 | -7.339 | 1 | 32.877 | 0 | 0 |
| Flavobacteriaceae | Ulvibacter | NA | 16.79 | -7.85 | 0.869 | 17.871 | 0 | 0 |
| Flavobacteriaceae | Jejudonia | NA | 8.725 | -2.268 | 0.834 | 7.048 | 0.008 | 0.038 |
| Flavobacteriaceae | Kordia | NA | 29.596 | 6.553 | 1.169 | 15.66 | 0 | 0.001 |
| Flavobacteriaceae | Kordia | jejudonensis | 84.147 | 5.963 | 0.578 | 66.903 | 0 | 0 |
| Flavobacteriaceae | Kordia | NA | 6.941 | 4.927 | 1.165 | 12.53 | 0 | 0.003 |
| Flavobacteriaceae | Polaribacter | NA | 126.711 | 8.478 | 0.705 | 79.666 | 0 | 0 |
| Flavobacteriaceae | NA | NA | 52.243 | -5.064 | 1.029 | 6.894 | 0.009 | 0.041 |
| Flavobacteriaceae | Aurantivirga | NA | 28.861 | 9.283 | 1.085 | 16.011 | 0 | 0.001 |
| Cryomorphaceae | NS10_marine_group | NA | 22.929 | 24.533 | 1.461 | 7.308 | 0.007 | 0.034 |
| Crocinitomicaceae | Crocinitomix | NA | 19.245 | 6.306 | 0.883 | 27.094 | 0 | 0 |
| Crocinitomicaceae | NA | NA | 6.864 | 4.854 | 1.422 | 6.997 | 0.008 | 0.039 |
| Schleiferiaceae | Schleiferia | NA | 3.247 | 4.559 | 1.404 | 8.77 | 0.003 | 0.019 |
| 37-13 | NA | NA | 3.47 | 3.065 | 0.937 | 8.643 | 0.003 | 0.019 |
| Saprospiraceae | Phaeodactylibacter | NA | 5.174 | 4.593 | 1.116 | 13.229 | 0 | 0.002 |
| Saprospiraceae | NA | NA | 31.823 | 4.771 | 1.005 | 14.881 | 0 | 0.001 |
| Saprospiraceae | NA | NA | 3.277 | 3.567 | 1.432 | 6.474 | 0.011 | 0.049 |
| Saprospiraceae | Lewinella | NA | 7.277 | 4.275 | 0.952 | 13.473 | 0 | 0.002 |
| Saprospiraceae | Lewinella | NA | 12.896 | 3.2 | 0.82 | 12.708 | 0 | 0.003 |
| Saprospiraceae | NA | NA | 9.241 | -3.13 | 0.727 | 8.447 | 0.004 | 0.021 |
| Saprospiraceae | Lewinella | persica | 23.037 | 3.204 | 0.555 | 21.879 | 0 | 0 |
| Saprospiraceae | NA | NA | 16.243 | 2.036 | 1.061 | 8.7 | 0.003 | 0.019 |
| Saprospiraceae | NA | NA | 7.088 | 5.254 | 1.081 | 17.731 | 0 | 0 |
| Saprospiraceae | NA | NA | 5.406 | 5.217 | 1.471 | 9.965 | 0.002 | 0.011 |
| Saprospiraceae | NA | NA | 4.064 | 3.836 | 1.055 | 11.465 | 0.001 | 0.005 |
| Saprospiraceae | Portibacter | NA | 34.143 | 2.237 | 0.531 | 13.556 | 0 | 0.002 |
| Saprospiraceae | Portibacter | NA | 14.035 | 2.413 | 0.754 | 6.636 | 0.01 | 0.046 |
| Saprospiraceae | NA | NA | 14.664 | 5.055 | 0.685 | 41.695 | 0 | 0 |
| Saprospiraceae | NA | NA | 40.698 | 2.5 | 0.581 | 12.285 | 0 | 0.004 |
| Saprospiraceae | NA | NA | 15.48 | 3.791 | 1.066 | 7.784 | 0.005 | 0.028 |
| Saprospiraceae | NA | NA | 5.978 | 4.909 | 1.177 | 12.193 | 0 | 0.004 |
| Saprospiraceae | NA | NA | 14.502 | 4.283 | 1.205 | 8.618 | 0.003 | 0.02 |
| Saprospiraceae | NA | NA | 25.68 | 5.793 | 1.522 | 7.916 | 0.005 | 0.027 |
| Saprospiraceae | NA | NA | 19.903 | 5.377 | 0.865 | 18.326 | 0 | 0 |
| Saprospiraceae | NA | NA | 16.506 | 6.402 | 0.908 | 33.039 | 0 | 0 |
| Saprospiraceae | Rubidimonas | NA | 19.148 | 4.872 | 1.562 | 7.218 | 0.007 | 0.036 |
| Saprospiraceae | Rubidimonas | NA | 46.119 | 5.803 | 0.735 | 32.33 | 0 | 0 |
| Saprospiraceae | Rubidimonas | NA | 16.484 | 6.004 | 0.837 | 34.507 | 0 | 0 |
| Saprospiraceae | NA | NA | 8.404 | 2.714 | 0.828 | 9.139 | 0.003 | 0.016 |
| Saprospiraceae | NA | NA | 9.067 | 4.217 | 1.034 | 8.181 | 0.004 | 0.024 |
| Cryomorphaceae | Vicingus | NA | 8.809 | 3.477 | 1.132 | 7.415 | 0.006 | 0.033 |
| Cryomorphaceae | Vicingus | NA | 39.92 | 8.815 | 1.106 | 15.058 | 0 | 0.001 |
| NS9_marine_group | NA | NA | 7.303 | 5.443 | 1.099 | 14.389 | 0 | 0.002 |
| Crocinitomicaceae | NA | NA | 11.541 | 5.341 | 1.069 | 15.945 | 0 | 0.001 |
| Flavobacteriaceae | NA | NA | 4.78 | -4.952 | 0.905 | 25.497 | 0 | 0 |
| Flavobacteriaceae | Maribacter | NA | 60.234 | -1.936 | 0.44 | 12.808 | 0 | 0.003 |
| Flavobacteriaceae | Maribacter | NA | 27.545 | -1.906 | 0.648 | 6.519 | 0.011 | 0.048 |
| Flavobacteriaceae | Robiginitalea | NA | 19.83 | -1.515 | 0.491 | 7.561 | 0.006 | 0.031 |
| Cryomorphaceae | NA | NA | 5.3 | 2.918 | 0.929 | 6.955 | 0.008 | 0.04 |
| Cryomorphaceae | NA | NA | 11.39 | 2.924 | 0.771 | 12.212 | 0 | 0.004 |
| Crocinitomicaceae | Fluviicola | NA | 8.382 | 4.419 | 0.965 | 15.854 | 0 | 0.001 |
| Crocinitomicaceae | Fluviicola | NA | 59.221 | 4.42 | 0.631 | 30.869 | 0 | 0 |
| Crocinitomicaceae | Fluviicola | NA | 13.2 | 5.254 | 1.068 | 13.396 | 0 | 0.002 |
| Cryomorphaceae | NA | NA | 10.5 | 4.733 | 0.759 | 27.193 | 0 | 0 |
| Cryomorphaceae | NA | NA | 2.929 | 3.702 | 1.276 | 6.579 | 0.01 | 0.047 |
| NS11-12_marine_group | NA | NA | 9.853 | 4.851 | 1.393 | 9.401 | 0.002 | 0.014 |
| Caldilineaceae | NA | NA | 18.228 | 2.314 | 0.763 | 7.962 | 0.005 | 0.026 |
| Rubritaleaceae | Roseibacillus | NA | 11.684 | -3.547 | 0.997 | 10.048 | 0.002 | 0.011 |
| Ilumatobacteraceae | Ilumatobacter | nonamiensis | 11.214 | -2.746 | 0.78 | 7.476 | 0.006 | 0.032 |
| Microtrichaceae | Sva0996_marine_group | NA | 9.877 | -2.022 | 0.701 | 6.627 | 0.01 | 0.046 |
| Microtrichaceae | NA | NA | 16.752 | 2.333 | 0.62 | 8.951 | 0.003 | 0.017 |
| Microtrichaceae | Sva0996_marine_group | NA | 10.17 | 5.125 | 0.904 | 8.315 | 0.004 | 0.022 |
| Microtrichaceae | Sva0996_marine_group | NA | 6.494 | -4.211 | 1.047 | 11.61 | 0.001 | 0.005 |
| Bdellovibrionaceae | OM27_clade | NA | 2.785 | 3.614 | 1.084 | 9.361 | 0.002 | 0.014 |
| Desulfobulbaceae | Desulfobulbus | NA | 10.12 | -2.364 | 0.712 | 7.388 | 0.007 | 0.033 |
| Magnetospiraceae | NA | NA | 3.245 | 3.085 | 0.955 | 7 | 0.008 | 0.039 |
| Terasakiellaceae | NA | NA | 20.178 | 5.687 | 0.858 | 29.384 | 0 | 0 |
| Rhodobacteraceae | Thiobacimonas | NA | 23.578 | -1.724 | 0.454 | 13.363 | 0 | 0.002 |
| Hyphomicrobiaceae | Filomicrobium | NA | 14.015 | -3.249 | 0.556 | 22.389 | 0 | 0 |
| Hyphomonadaceae | Hyphomonas | NA | 6.878 | -3.616 | 0.796 | 13.309 | 0 | 0.002 |
| Hyphomonadaceae | NA | NA | 17.565 | -1.767 | 0.541 | 8.435 | 0.004 | 0.021 |
| Hyphomonadaceae | Hellea | balneolensis | 32.065 | 3.115 | 0.42 | 42.073 | 0 | 0 |
| Hyphomonadaceae | Litorimonas | NA | 19.341 | 3.782 | 0.691 | 19.64 | 0 | 0 |
| Hyphomonadaceae | Robiginitomaculum | NA | 9.688 | 2.369 | 0.773 | 8.735 | 0.003 | 0.019 |
| Rhodobacteraceae | NA | NA | 13.374 | 3.927 | 0.774 | 17.581 | 0 | 0 |
| Rhodobacteraceae | NA | NA | 156.8 | 5.239 | 0.369 | 116.098 | 0 | 0 |
| Rhodobacteraceae | NA | NA | 26.224 | 6.988 | 0.746 | 35.258 | 0 | 0 |
| Rhodobacteraceae | NA | NA | 49.826 | 5.501 | 0.611 | 50.96 | 0 | 0 |
| Rhodobacteraceae | Yoonia-Loktanella | NA | 9.386 | 4.7 | 1.075 | 11.11 | 0.001 | 0.006 |
| Rhodobacteraceae | Jannaschia | NA | 18.178 | -6.647 | 0.856 | 34.293 | 0 | 0 |
| Rhodobacteraceae | NA | NA | 13.839 | 2.533 | 0.813 | 8.737 | 0.003 | 0.019 |
| Rhodobacteraceae | NA | NA | 11.039 | -5.772 | 0.912 | 12.84 | 0 | 0.003 |
| Rhodobacteraceae | Octadecabacter | NA | 134.373 | 4.597 | 0.722 | 24.126 | 0 | 0 |
| Rhodobacteraceae | Octadecabacter | NA | 182.503 | -1.684 | 0.26 | 34.455 | 0 | 0 |
| Rhodobacteraceae | Tropicimonas | NA | 3.973 | -3.845 | 1.326 | 8.573 | 0.003 | 0.02 |
| Rhodobacteraceae | Planktotalea | NA | 188.23 | 0.797 | 0.285 | 7.221 | 0.007 | 0.036 |
| Rhodobacteraceae | Litoreibacter | NA | 23.105 | -6.355 | 0.984 | 10.752 | 0.001 | 0.008 |
| Rhodobacteraceae | Aliiroseovarius | NA | 30.58 | 7.517 | 1.029 | 10.494 | 0.001 | 0.009 |
| Rhodobacteraceae | Celeribacter | NA | 45.875 | -2.037 | 0.391 | 23.139 | 0 | 0 |
| Rhodobacteraceae | NA | NA | 28.868 | 3.607 | 0.712 | 15.82 | 0 | 0.001 |
| Rhodobacteraceae | NA | NA | 297.305 | 1.386 | 0.23 | 31.552 | 0 | 0 |
| Rhodobacteraceae | NA | NA | 23.106 | 7.592 | 0.722 | 71.166 | 0 | 0 |
| Rhodobacteraceae | NA | NA | 70.863 | 2.018 | 0.396 | 20.735 | 0 | 0 |
| Rhodobacteraceae | Pacificibacter | marinus | 13.612 | 2.118 | 0.778 | 7.438 | 0.006 | 0.033 |
| Rhodobacteraceae | NA | NA | 7.986 | -2.647 | 1.016 | 6.769 | 0.009 | 0.043 |
| Rhodobacteraceae | NA | NA | 14.836 | 6.254 | 1.052 | 20.536 | 0 | 0 |
| Rhodobacteraceae | Sulfitobacter | litoralis | 67.491 | -1.478 | 0.35 | 13.263 | 0 | 0.002 |
| Rhodobacteraceae | Sulfitobacter | NA | 31.37 | 27.294 | 1.415 | 10.834 | 0.001 | 0.007 |
| Rhodobacteraceae | Sulfitobacter | NA | 8.443 | -4.069 | 1.083 | 8.749 | 0.003 | 0.019 |
| Rhodobacteraceae | Sulfitobacter | NA | 46.035 | 2.743 | 0.806 | 6.605 | 0.01 | 0.046 |
| Rhodobacteraceae | Roseobacter | litoralis | 50.7 | -1.681 | 0.545 | 7.315 | 0.007 | 0.034 |
| Rhodobacteraceae | Sedimentitalea | NA | 30.39 | -5.095 | 0.721 | 32.182 | 0 | 0 |
| Rhodobacteraceae | NA | NA | 6.984 | -9.575 | 1.238 | 9.181 | 0.002 | 0.015 |
| Rhodobacteraceae | Phaeobacter | NA | 17.936 | -6.293 | 0.781 | 22.546 | 0 | 0 |
| Rhodobacteraceae | NA | NA | 26.276 | 5.002 | 0.747 | 23.799 | 0 | 0 |
| Rhodobacteraceae | Roseovarius | aestuarii | 8.958 | -5.594 | 1.01 | 24.136 | 0 | 0 |
| Rhodobacteraceae | NA | NA | 9.473 | 5.571 | 1.118 | 13.972 | 0 | 0.002 |
| Rhodobacteraceae | NA | NA | 23.751 | 3.868 | 0.534 | 39.091 | 0 | 0 |
| Rhodobacteraceae | Sedimentitalea | NA | 55.962 | 3.298 | 0.457 | 35.005 | 0 | 0 |
| Rhodobacteraceae | NA | NA | 6.103 | -4.458 | 0.794 | 26.924 | 0 | 0 |
| Rhodobacteraceae | NA | NA | 27.698 | 4.835 | 0.552 | 48.63 | 0 | 0 |
| Rhodobacteraceae | Yoonia-Loktanella | NA | 34.729 | 2.619 | 0.81 | 7.806 | 0.005 | 0.028 |
| Rhodobacteraceae | NA | NA | 54.699 | 2.74 | 0.433 | 30.576 | 0 | 0 |
| Rhodobacteraceae | NA | NA | 22.331 | 6.23 | 1.218 | 14.552 | 0 | 0.001 |
| Rhodobacteraceae | NA | NA | 38.101 | 2.069 | 0.45 | 13.874 | 0 | 0.002 |
| Rhodobacteraceae | Leisingera | NA | 6.599 | -3.377 | 1.067 | 9.945 | 0.002 | 0.011 |
| Rhodobacteraceae | Yoonia-Loktanella | NA | 28.841 | 2.622 | 0.596 | 12.907 | 0 | 0.003 |
| Rhodobacteraceae | Yoonia-Loktanella | NA | 33.033 | 1.722 | 0.361 | 19.916 | 0 | 0 |
| Rhodobacteraceae | NA | NA | 91.674 | 2.711 | 0.319 | 56.853 | 0 | 0 |
| Rhizobiaceae | Pseudahrensia | NA | 16.213 | 3.444 | 0.764 | 12.287 | 0 | 0.004 |
| Rhizobiaceae | Pseudahrensia | NA | 33.824 | -2.058 | 0.528 | 9.676 | 0.002 | 0.012 |
| Rhizobiaceae | Pseudahrensia | NA | 20.369 | -6.875 | 0.646 | 82.222 | 0 | 0 |
| Rhodobacteraceae | NA | NA | 5.136 | -4.7 | 0.936 | 20.204 | 0 | 0 |
| Rhodobacteraceae | NA | NA | 52.283 | -2.369 | 0.365 | 34.517 | 0 | 0 |
| Rhizobiaceae | Pseudahrensia | NA | 12.006 | -6.025 | 0.694 | 25.431 | 0 | 0 |
| Rhizobiaceae | Hoeflea | NA | 6.32 | -3.331 | 1.187 | 7.573 | 0.006 | 0.031 |
| Sphingomonadaceae | NA | NA | 33.438 | -2.647 | 0.722 | 11.976 | 0.001 | 0.004 |
| Saprospiraceae | NA | NA | 42.309 | 6.654 | 0.899 | 31.117 | 0 | 0 |
| Spirosomaceae | Taeseokella | NA | 38.22 | 3.031 | 0.441 | 37.073 | 0 | 0 |
| Flavobacteriaceae | NS3a_marine_group | NA | 84.418 | 3.371 | 0.772 | 8.954 | 0.003 | 0.017 |
| Rhodothermaceae | NA | NA | 20.236 | 2.904 | 0.477 | 24.9 | 0 | 0 |
| Desulfosarcinaceae | Sva0081_sediment_group | NA | 4.345 | -3.204 | 1.302 | 6.871 | 0.009 | 0.041 |
| Desulfocapsaceae | NA | NA | 5.959 | 5.338 | 1.447 | 8.979 | 0.003 | 0.017 |
| Desulfocapsaceae | NA | NA | 18.038 | -2.535 | 0.654 | 9.831 | 0.002 | 0.012 |
| Desulfocapsaceae | NA | NA | 5.066 | -3.818 | 1.333 | 9.487 | 0.002 | 0.014 |
| Desulfocapsaceae | NA | NA | 64.664 | -1.233 | 0.36 | 10.256 | 0.001 | 0.01 |
| Desulfocapsaceae | NA | NA | 66.622 | -1.211 | 0.351 | 10.572 | 0.001 | 0.008 |
| Desulfocapsaceae | NA | NA | 18.128 | -2.126 | 0.538 | 12.336 | 0 | 0.004 |
| Fokiniaceae | MD3-55 | NA | 11.348 | 5.372 | 0.572 | 65.274 | 0 | 0 |
| Rhizobiales_Incertae_Sedis | Anderseniella | NA | 2.796 | -3.935 | 1.288 | 8.48 | 0.004 | 0.021 |
| Rhizobiaceae | NA | NA | 4.211 | -4.969 | 1.607 | 8.83 | 0.003 | 0.018 |
| Rhizobiaceae | NA | NA | 8.682 | -2.859 | 0.806 | 10.624 | 0.001 | 0.008 |
| Rhodobacteraceae | Tateyamaria | NA | 77.176 | 3.393 | 0.416 | 45.07 | 0 | 0 |
| Rhodobacteraceae | Tateyamaria | NA | 11.514 | -8.193 | 1.125 | 11.903 | 0.001 | 0.004 |
| Rhodobacteraceae | NA | NA | 23.077 | 4.569 | 0.575 | 40.098 | 0 | 0 |
| Rhizobiaceae | Ahrensia | NA | 4.859 | -4.253 | 1.154 | 10.378 | 0.001 | 0.009 |
| Sphingomonadaceae | Parasphingopyxis | NA | 11.706 | -5.257 | 0.837 | 23.948 | 0 | 0 |
| Micavibrionaceae | NA | NA | 5.976 | 3.818 | 1.049 | 7.985 | 0.005 | 0.026 |
| Micavibrionaceae | NA | NA | 3.442 | -4.79 | 1.156 | 16.063 | 0 | 0.001 |
| Micavibrionaceae | NA | NA | 11.927 | -3.664 | 0.9 | 13.377 | 0 | 0.002 |
| Rickettsiaceae | Candidatus_Megaira | NA | 41.926 | 6.587 | 0.791 | 39.498 | 0 | 0 |

**Table S4**

| **Pathway** | **log2-fold Change** |
| --- | --- |
| nitrifier denitrification | 3.57517042 |
| superpathway of polyamine biosynthesis III | 2.81827993 |
| CMP-pseudaminate biosynthesis | 2.62893542 |
| nylon-6 oligomer degradation | 1.64149213 |
| formaldehyde oxidation I | 1.19448623 |
| formaldehyde assimilation II (RuMP Cycle) | 1.18703389 |
| thiazole biosynthesis II (Bacillus) | 1.17319111 |
| coenzyme M biosynthesis I | 1.06840996 |
| superpathway of thiamin diphosphate biosynthesis II | 0.98492463 |
| methyl ketone biosynthesis | 0.93025842 |
| L-arginine degradation II (AST pathway) | 0.87076592 |
| glucose and glucose-1-phosphate degradation | 0.71012766 |
| ectoine biosynthesis | 0.68464435 |
| norspermidine biosynthesis | 0.59741725 |
| ADP-L-glycero-&beta;-D-manno-heptose biosynthesis | 0.59480138 |
| superpathway of polyamine biosynthesis I | 0.50117322 |
| catechol degradation II (meta-cleavage pathway) | -0.5051274 |
| L-tryptophan degradation XII (Geobacillus) | -0.5311326 |
| catechol degradation I (meta-cleavage pathway) | -0.5785306 |
| acetylene degradation | -0.599038 |
| 2-aminophenol degradation | -0.6086563 |
| catechol degradation to &beta;-ketoadipate | -0.6123384 |
| superpathway of pyridoxal 5'-phosphate biosynthesis and salvage | -0.6160838 |
| superpathway of sulfur oxidation (Acidianus ambivalens) | -0.7083875 |
| reductive acetyl coenzyme A pathway | -0.8598703 |
| meta cleavage pathway of aromatic compounds | -0.8753625 |
| adenosylcobalamin biosynthesis II (late cobalt incorporation) | -0.9123838 |
| androstenedione degradation | -0.9491612 |
| superpathway of salicylate degradation | -0.9500477 |
| methanogenesis from acetate | -0.9597901 |
| catechol degradation III (ortho-cleavage pathway) | -0.968522 |
| aromatic compounds degradation via &beta;-ketoadipate | -0.968522 |
| formaldehyde assimilation I (serine pathway) | -0.9774884 |
| superpathway of 2,3-butanediol biosynthesis | -0.9804931 |
| D-galactarate degradation I | -1.0428591 |
| superpathway of D-glucarate and D-galactarate degradation | -1.0428591 |
| pyruvate fermentation to acetone | -1.0446446 |
| isopropanol biosynthesis | -1.0943399 |
| superpathway of (R,R)-butanediol biosynthesis | -1.1560577 |
| superpathway of L-aspartate and L-asparagine biosynthesis | -1.2056707 |
| glycerol degradation to butanol | -1.2387094 |
| superpathway of N-acetylneuraminate degradation | -1.2987337 |
| superpathway of N-acetylglucosamine, N-acetylmannosamine and N-acetylneuraminate degradation | -1.407782 |
| creatinine degradation II | -1.4875672 |
| D-glucarate degradation I | -1.4888527 |
| 1,5-anhydrofructose degradation | -1.5852009 |
| allantoin degradation to glyoxylate III | -1.6402326 |
| mono-trans, poly-cis decaprenyl phosphate biosynthesis | -1.6413193 |
| cob(II)yrinate a,c-diamide biosynthesis I (early cobalt insertion) | -2.4457325 |
| methylaspartate cycle | -2.4547742 |
| coenzyme B biosynthesis | -2.718622 |
| chondroitin sulfate degradation I (bacterial) | -3.8564041 |
| starch degradation III | -5.3764083 |

**Table S5**

|  |  |  | df | Sum Of Squares | R^2^ | F-Statistic | Pr(>F) |
| --- | --- | --- | --- | --- | --- | --- | --- |
| Based on taxonomy | Root vs Mimic | Sample Type | 1 | 4716.5 | 0.30739 | 31.511 | **0.001** |
|  |  | Residual | 71 | 10627.2 | 0.69261 |  |  |
|  |  | Total | 72 | 15343.7 | 1 |  |  |
|  | Mimic vs. Sediment | Sample Type | 1 | 5659.8 | 0.42429 | 44.22 | **0.001** |
|  |  | Residual | 60 | 7679.5 | 0.57571 |  |  |
|  |  | Total | 61 | 13339.3 | 1 |  |  |
|  | Root vs. Sediment | Sample Type | 1 | 3234.5 | 0.24806 | 26.722 | **0.001** |
|  |  | Residual | 81 | 9804.5 | 0.75194 |  |  |
|  |  | Total | 82 | 13039.1 | 1 |  |  |
| Based on predicted function | Root vs Mimic | Sample Type | 1 | 4991 | 0.11386 | 9.1228 | **0.001** |
|  |  | Residual | 71 | 38841 | 0.88614 |  |  |
|  |  | Total | 72 | 43831 | 1 |  |  |
|  | Mimic vs. Sediment | Sample Type | 1 | 8250 | 0.19562 | 14.592 | **0.001** |
|  |  | Residual | 60 | 33924 | 0.80438 |  |  |
|  |  | Total | 61 | 42174 | 1 |  |  |
|  | Root vs. Sediment | Sample Type | 1 | 4865 | 0.1482 | 14.093 | **0.001** |
|  |  | Residual | 81 | 27963 | 0.8518 |  |  |
|  |  | Total | 82 | 32829 | 1 |  |  |

**Table S6**

| **Family** | **Higher on roots** | **Higher on mimics** | **Higher on mimics** | **Higher on sediment** | **Higher on roots** | **Higher on sediment** |
| --- | --- | --- | --- | --- | --- | --- |
| 4572-13 | 3 | 0 | 0 | 2 | 1 | 2 |
| Acanthopleuribacteraceae | 2 | 0 | 0 | 1 | 1 | 1 |
| Anaerolineaceae | 12 | 0 | 1 | 11 | 3 | 10 |
| Arcobacteraceae | 2 | 0 | 1 | 0 | 2 | 0 |
| Arenicellaceae | 0 | 1 | 0 | 1 | 0 | 1 |
| Bacteroidetes_BD2-2 | 31 | 1 | 0 | 25 | 16 | 14 |
| Calditrichaceae | 10 | 1 | 0 | 11 | 1 | 10 |
| Cellvibrionaceae | 1 | 0 | 1 | 0 | 1 | 0 |
| Christensenellaceae | 3 | 0 | 0 | 3 | 2 | 1 |
| Chromatiaceae | 4 | 0 | 0 | 4 | 0 | 4 |
| Crocinitomicaceae | 1 | 0 | 2 | 0 | 2 | 0 |
| Cyclobacteriaceae | 3 | 0 | 0 | 3 | 0 | 3 |
| Desulfatiglandaceae | 4 | 0 | 0 | 4 | 0 | 4 |
| Desulfobacteraceae | 9 | 0 | 0 | 5 | 7 | 2 |
| Desulfobulbaceae | 8 | 0 | 1 | 7 | 4 | 5 |
| Desulfocapsaceae | 40 | 0 | 8 | 25 | 27 | 9 |
| Desulfococcaceae | 1 | 0 | 0 | 1 | 1 | 0 |
| Desulfolunaceae | 1 | 0 | 0 | 1 | 0 | 1 |
| Desulfomonilaceae | 0 | 1 | 0 | 1 | 0 | 1 |
| Desulfosarcinaceae | 34 | 0 | 0 | 35 | 8 | 26 |
| Desulfovibrionaceae | 3 | 0 | 0 | 1 | 3 | 0 |
| Ectothiorhodospiraceae | 1 | 1 | 0 | 2 | 0 | 2 |
| Fermentibacteraceae | 3 | 0 | 0 | 3 | 1 | 2 |
| Fibrobacteraceae | 1 | 0 | 0 | 1 | 1 | 0 |
| Flavobacteriaceae | 24 | 13 | 19 | 20 | 21 | 17 |
| Fusibacteraceae | 1 | 0 | 0 | 1 | 1 | 0 |
| Gemmatimonadaceae | 1 | 0 | 0 | 1 | 0 | 1 |
| Geopsychrobacteraceae | 2 | 0 | 1 | 0 | 2 | 0 |
| Halieaceae | 9 | 0 | 1 | 9 | 1 | 9 |
| Halomonadaceae | 0 | 2 | 2 | 0 | 2 | 0 |
| Hungateiclostridiaceae | 3 | 0 | 0 | 3 | 1 | 2 |
| Hyphomonadaceae | 0 | 2 | 2 | 0 | 2 | 0 |
| Ignavibacteriaceae | 1 | 0 | 0 | 1 | 0 | 1 |
| Kiritimatiellaceae | 3 | 1 | 0 | 3 | 2 | 1 |
| Lachnospiraceae | 8 | 0 | 3 | 3 | 8 | 0 |
| Latescibacteraceae | 1 | 0 | 0 | 3 | 0 | 3 |
| Lentimicrobiaceae | 7 | 0 | 0 | 7 | 1 | 3 |
| Leptospiraceae | 1 | 0 | 0 | 1 | 1 | 0 |
| Marinifilaceae | 6 | 0 | 0 | 2 | 6 | 0 |
| Marinilabiliaceae | 9 | 0 | 0 | 8 | 6 | 3 |
| Marinomonadaceae | 1 | 0 | 1 | 0 | 1 | 0 |
| Melioribacteraceae | 7 | 0 | 0 | 6 | 3 | 4 |
| Methylophagaceae | 2 | 0 | 1 | 1 | 2 | 0 |
| Methylophilaceae | 1 | 0 | 2 | 0 | 2 | 0 |
| Moduliflexaceae | 19 | 0 | 0 | 14 | 16 | 3 |
| MSBL8 | 5 | 0 | 1 | 4 | 2 | 2 |
| Nitrincolaceae | 1 | 0 | 0 | 1 | 1 | 0 |
| NS11-12_marine_group | 0 | 1 | 1 | 0 | 1 | 0 |
| Pedosphaeraceae | 1 | 0 | 0 | 1 | 0 | 1 |
| PHOS-HE36 | 2 | 0 | 0 | 3 | 0 | 3 |
| Pirellulaceae | 9 | 1 | 2 | 10 | 2 | 9 |
| Prolixibacteraceae | 16 | 0 | 1 | 11 | 10 | 3 |
| Puniceicoccaceae | 2 | 0 | 1 | 0 | 2 | 0 |
| Rhizobiaceae | 4 | 0 | 3 | 0 | 5 | 0 |
| Rhodobacteraceae | 5 | 4 | 17 | 0 | 17 | 0 |
| Rickettsiaceae | 1 | 0 | 1 | 0 | 1 | 0 |
| Rubinisphaeraceae | 0 | 1 | 2 | 0 | 2 | 0 |
| S15A-MN91 | 1 | 0 | 0 | 1 | 1 | 0 |
| Sandaracinaceae | 1 | 0 | 0 | 1 | 0 | 1 |
| Saprospiraceae | 5 | 5 | 6 | 5 | 6 | 3 |
| SB-5 | 11 | 0 | 0 | 8 | 6 | 5 |
| Sedimenticolaceae | 8 | 2 | 1 | 9 | 4 | 6 |
| SG8-4 | 3 | 0 | 0 | 2 | 1 | 2 |
| Shewanellaceae | 0 | 1 | 1 | 0 | 1 | 0 |
| Spirochaetaceae | 23 | 0 | 1 | 21 | 15 | 11 |
| Spirosomaceae | 0 | 1 | 1 | 0 | 1 | 0 |
| Spongiibacteraceae | 2 | 1 | 2 | 1 | 2 | 1 |
| Sulfurimonadaceae | 6 | 1 | 1 | 1 | 6 | 1 |
| Sulfurovaceae | 1 | 0 | 1 | 0 | 1 | 0 |
| Syntrophotaleaceae | 1 | 0 | 0 | 1 | 1 | 0 |
| Thermoanaerobaculaceae | 13 | 0 | 0 | 14 | 0 | 14 |
| Thioalkalispiraceae | 1 | 1 | 0 | 4 | 0 | 4 |
| Thiohalorhabdaceae | 1 | 0 | 0 | 1 | 0 | 1 |
| Thiomicrospiraceae | 7 | 2 | 0 | 9 | 1 | 8 |
| Thiotrichaceae | 2 | 5 | 5 | 4 | 5 | 4 |
| Unknown_Family | 9 | 0 | 1 | 10 | 1 | 10 |
| Vibrionaceae | 1 | 0 | 1 | 0 | 1 | 0 |
| Woeseiaceae | 3 | 0 | 0 | 3 | 0 | 3 |

**Table S7**

See excel file

**Table S8**

| **Pathway** | **Root vs. Mimic** | **Mimic vs. Sediment** | **Root vs. Sediment** |
| --- | --- | --- | --- |
| &beta;-alanine biosynthesis II | -2.707 | 6.111 | 3.404 |
| 1,4-dihydroxy-2-naphthoate biosynthesis I | -0.734 | NA | NA |
| 1,4-dihydroxy-6-naphthoate biosynthesis I | 0.895 | -0.833 | NA |
| 1,4-dihydroxy-6-naphthoate biosynthesis II | 0.936 | -0.922 | NA |
| 2-amino-3-carboxymuconate semialdehyde degradation to 2-oxopentenoate | -1.392 | 2.038 | 0.646 |
| 2-aminophenol degradation | -2.279 | 1.971 | NA |
| 2-methylcitrate cycle I | -1.101 | NA | -0.666 |
| 2-methylcitrate cycle II | -0.814 | NA | -0.5 |
| 2-nitrobenzoate degradation I | -1.351 | 1.919 | 0.568 |
| 3-phenylpropanoate and 3-(3-hydroxyphenyl)propanoate degradation | -1.066 | 3.175 | 2.11 |
| 3-phenylpropanoate degradation | -2.55 | 7.214 | 4.664 |
| 4-coumarate degradation (anaerobic) | NA | 0.894 | 0.511 |
| 4-deoxy-L-threo-hex-4-enopyranuronate degradation | NA | NA | 0.568 |
| 4-hydroxyphenylacetate degradation | -0.697 | 1.697 | 1 |
| 4-methylcatechol degradation (ortho cleavage) | -2.808 | 2.565 | NA |
| acetylene degradation | NA | NA | 0.599 |
| adenosylcobalamin biosynthesis I (early cobalt insertion) | -0.714 | 2.256 | 1.542 |
| adenosylcobalamin biosynthesis II (late cobalt incorporation) | -0.868 | 2.381 | 1.512 |
| ADP-L-glycero-&beta;-D-manno-heptose biosynthesis | 0.625 | -1.079 | NA |
| aerobactin biosynthesis | -0.913 | 2.622 | 1.709 |
| allantoin degradation IV (anaerobic) | -5.19 | 12.389 | 7.199 |
| allantoin degradation to glyoxylate III | -1.345 | 1.089 | NA |
| androstenedione degradation | NA | -1.1 | -1.165 |
| arginine, ornithine and proline interconversion | 0.831 | NA | 0.59 |
| aromatic biogenic amine degradation (bacteria) | -0.658 | 0.859 | NA |
| aromatic compounds degradation via &beta;-ketoadipate | -2.45 | 2.489 | NA |
| benzoyl-CoA degradation II (anaerobic) | 2.335 | -2.511 | NA |
| Bifidobacterium shunt | -1.207 | 1.348 | NA |
| biotin biosynthesis II | -1.18 | 5.052 | 3.871 |
| catechol degradation I (meta-cleavage pathway) | NA | NA | -0.548 |
| catechol degradation III (ortho-cleavage pathway) | -2.45 | 2.489 | NA |
| catechol degradation to &beta;-ketoadipate | -1.833 | 2.268 | NA |
| catechol degradation to 2-oxopent-4-enoate II | -0.686 | 1.26 | 0.574 |
| chitin derivatives degradation | NA | 0.947 | 1.405 |
| chlorophyllide a biosynthesis I (aerobic, light-dependent) | -0.72 | 1.652 | 0.932 |
| chlorophyllide a biosynthesis II (anaerobic) | -0.768 | 1.592 | 0.823 |
| chlorophyllide a biosynthesis III (aerobic, light independent) | -0.768 | 1.592 | 0.823 |
| chlorosalicylate degradation | -3.061 | 5.32 | 2.259 |
| chondroitin sulfate degradation I (bacterial) | -1.706 | 2.696 | 0.99 |
| CMP-legionaminate biosynthesis I | 0.934 | -1.577 | -0.643 |
| CMP-pseudaminate biosynthesis | 2.677 | 3.179 | 5.856 |
| cob(II)yrinate a,c-diamide biosynthesis I (early cobalt insertion) | NA | 1.762 | 1.522 |
| cob(II)yrinate a,c-diamide biosynthesis II (late cobalt incorporation) | -0.532 | 1.015 | NA |
| coenzyme B biosynthesis | -3.071 | 6.334 | 3.263 |
| coenzyme M biosynthesis I | -0.653 | NA | NA |
| creatinine degradation I | -0.731 | 1.411 | 0.68 |
| creatinine degradation II | -1.043 | 1.966 | 0.923 |
| D-fructuronate degradation | -0.69 | 0.755 | NA |
| D-galactarate degradation I | -1.061 | 0.528 | -0.533 |
| D-galacturonate degradation I | NA | 0.635 | NA |
| D-glucarate degradation I | -1.669 | NA | -1.318 |
| dTDP-N-acetylthomosamine biosynthesis | -1.002 | 0.717 | NA |
| ectoine biosynthesis | -0.56 | 0.587 | NA |
| enterobacterial common antigen biosynthesis | -6.585 | 6.736 | NA |
| enterobactin biosynthesis | -2.12 | 1.54 | -0.58 |
| ergothioneine biosynthesis I (bacteria) | -5.705 | 4.133 | -1.572 |
| ethylmalonyl-CoA pathway | NA | 1.609 | 1.126 |
| factor 420 biosynthesis | -3.584 | 7.65 | 4.066 |
| formaldehyde assimilation I (serine pathway) | -1.108 | NA | -1.106 |
| formaldehyde assimilation II (RuMP Cycle) | -0.539 | 1.394 | 0.855 |
| formaldehyde oxidation I | -0.531 | 1.373 | 0.843 |
| galactose degradation I (Leloir pathway) | 0.553 | NA | NA |
| gallate degradation I | -1.414 | 2.933 | 1.519 |
| gallate degradation II | -1.449 | 2.968 | 1.52 |
| GDP-D-glycero-&alpha;-D-manno-heptose biosynthesis | 1.444 | -2.19 | -0.746 |
| glucose and glucose-1-phosphate degradation | -0.788 | 0.798 | NA |
| glucose degradation (oxidative) | -2.795 | 1.294 | -1.501 |
| glutaryl-CoA degradation | 0.7 | -1.314 | -0.614 |
| glycerol degradation to butanol | -1.06 | 1.965 | 0.905 |
| glycine betaine degradation I | NA | 1.479 | 1.012 |
| glycogen degradation I (bacterial) | 0.541 | -0.531 | NA |
| glycogen degradation II (eukaryotic) | -0.826 | 1.763 | 0.937 |
| glyoxylate cycle | -0.581 | 0.505 | NA |
| heterolactic fermentation | -1.206 | 1.333 | NA |
| hexitol fermentation to lactate, formate, ethanol and acetate | -3.151 | 3.158 | NA |
| incomplete reductive TCA cycle | 0.575 | -0.655 | NA |
| isoprene biosynthesis II (engineered) | 1.375 | -1.53 | NA |
| isopropanol biosynthesis | NA | -0.788 | -1.081 |
| ketogluconate metabolism | -2.08 | 2.904 | 0.823 |
| L-1,2-propanediol degradation | -3.483 | 7.5 | 4.017 |
| L-arabinose degradation IV | -1.091 | 10.119 | 9.027 |
| L-arginine degradation II (AST pathway) | -2.36 | 2.826 | NA |
| L-glutamate degradation V (via hydroxyglutarate) | 0.929 | -1.452 | -0.522 |
| L-histidine degradation II | -0.905 | 1.758 | 0.853 |
| L-isoleucine biosynthesis IV | 0.648 | -0.637 | NA |
| L-lysine biosynthesis II | -3.403 | 5.364 | 1.961 |
| L-lysine fermentation to acetate and butanoate | 0.561 | 1.344 | 1.905 |
| L-methionine biosynthesis I | NA | 0.651 | NA |
| L-methionine salvage cycle III | -6.548 | 9.365 | 2.817 |
| L-rhamnose degradation I | -0.601 | 0.575 | NA |
| L-tryptophan degradation IX | -0.755 | 0.943 | NA |
| L-tryptophan degradation to 2-amino-3-carboxymuconate semialdehyde | -1.013 | 0.945 | NA |
| L-tryptophan degradation XII (Geobacillus) | -1.826 | 1.525 | NA |
| L-tyrosine degradation I | -0.69 | 0.791 | NA |
| L-valine degradation I | -2.825 | 6.469 | 3.644 |
| lactose and galactose degradation I | -5.366 | 8.296 | 2.93 |
| mannan degradation | NA | 0.979 | 0.8 |
| meta cleavage pathway of aromatic compounds | -2.222 | 2.442 | NA |
| methanogenesis from acetate | 1.383 | -1.405 | NA |
| methanol oxidation to carbon dioxide | -1.267 | 1.652 | NA |
| methyl ketone biosynthesis | -0.595 | NA | -0.695 |
| methylaspartate cycle | NA | NA | 0.622 |
| methylgallate degradation | -1.426 | 2.944 | 1.518 |
| methylphosphonate degradation I | NA | 1.155 | 0.657 |
| mevalonate pathway I | 0.62 | -0.789 | NA |
| mevalonate pathway II (archaea) | 2.767 | -2.41 | NA |
| mono-trans, poly-cis decaprenyl phosphate biosynthesis | -4.413 | 7.054 | 2.641 |
| mycothiol biosynthesis | -1.242 | 0.619 | -0.623 |
| myo-, chiro- and scillo-inositol degradation | -1.407 | 2.468 | 1.061 |
| myo-inositol degradation I | -1.294 | 2.424 | 1.13 |
| NAD biosynthesis II (from tryptophan) | -0.799 | 0.725 | NA |
| NAD salvage pathway II | -3.019 | 2.894 | NA |
| nicotinate degradation I | -5.063 | 8.194 | 3.131 |
| nitrate reduction VI (assimilatory) | -1.019 | 1.286 | NA |
| nitrifier denitrification | -2.899 | 1.945 | -0.954 |
| norspermidine biosynthesis | -0.953 | 1.536 | 0.583 |
| nylon-6 oligomer degradation | -0.85 | 0.947 | NA |
| octane oxidation | -0.822 | 0.989 | NA |
| palmitate biosynthesis II (bacteria and plants) | NA | -0.905 | -1.09 |
| peptidoglycan biosynthesis II (staphylococci) | -5.146 | 11.401 | 6.254 |
| peptidoglycan biosynthesis IV (Enterococcus faecium) | -3.081 | 3.053 | NA |
| peptidoglycan biosynthesis V (&beta;-lactam resistance) | -3.874 | 6.005 | 2.131 |
| phenylacetate degradation I (aerobic) | -2.362 | 2.021 | NA |
| phospholipases | -1.595 | 1.442 | NA |
| polymyxin resistance | -3.01 | 1.664 | -1.346 |
| ppGpp biosynthesis | -0.775 | 1.179 | NA |
| protocatechuate degradation I (meta-cleavage pathway) | -1.35 | 3.374 | 2.025 |
| protocatechuate degradation II (ortho-cleavage pathway) | -0.963 | 1.364 | NA |
| purine nucleotides degradation II (aerobic) | NA | 0.761 | 0.626 |
| purine ribonucleosides degradation | NA | 0.974 | 1.037 |
| pyrimidine deoxyribonucleotides biosynthesis from CTP | 1.42 | -2.896 | -1.476 |
| pyrimidine deoxyribonucleotides de novo biosynthesis III | 0.531 | NA | NA |
| pyrimidine deoxyribonucleotides de novo biosynthesis IV | 1.396 | -2.894 | -1.498 |
| pyruvate fermentation to acetate and lactate II | 0.607 | NA | NA |
| pyruvate fermentation to acetone | -1.116 | NA | -0.677 |
| pyruvate fermentation to butanoate | 1.011 | -0.849 | NA |
| reductive acetyl coenzyme A pathway | 1.077 | -1.026 | NA |
| S-adenosyl-L-methionine cycle I | NA | 1.435 | 1.331 |
| S-methyl-5-thio-&alpha;-D-ribose 1-phosphate degradation | -6.882 | 9.53 | 2.648 |
| spirilloxanthin and 2,2'-diketo-spirilloxanthin biosynthesis | -0.97 | 2.188 | 1.218 |
| sucrose degradation II (sucrose synthase) | 0.736 | -2.09 | -1.354 |
| sucrose degradation III (sucrose invertase) | -1.619 | 2.032 | NA |
| superpathway of (Kdo)2-lipid A biosynthesis | -0.563 | -0.78 | -1.344 |
| superpathway of (R,R)-butanediol biosynthesis | -0.822 | NA | -1.035 |
| superpathway of &beta;-D-glucuronide and D-glucuronate degradation | -0.856 | 0.788 | NA |
| superpathway of 2,3-butanediol biosynthesis | -0.542 | NA | -0.89 |
| superpathway of aerobic toluene degradation | -1.618 | 1.714 | NA |
| superpathway of bacteriochlorophyll a biosynthesis | -0.717 | 1.655 | 0.938 |
| superpathway of C1 compounds oxidation to CO2 | NA | 3.717 | 3.424 |
| superpathway of chorismate metabolism | -1.082 | NA | -0.77 |
| superpathway of Clostridium acetobutylicum acidogenic fermentation | 0.964 | -0.791 | NA |
| superpathway of D-glucarate and D-galactarate degradation | -1.061 | 0.528 | -0.533 |
| superpathway of demethylmenaquinol-6 biosynthesis I | -0.559 | NA | NA |
| superpathway of demethylmenaquinol-6 biosynthesis II | 1.413 | 0.828 | 2.241 |
| superpathway of demethylmenaquinol-8 biosynthesis | -0.555 | NA | NA |
| superpathway of demethylmenaquinol-9 biosynthesis | -0.559 | NA | NA |
| superpathway of fucose and rhamnose degradation | -1.558 | 3.619 | 2.061 |
| superpathway of geranylgeranyldiphosphate biosynthesis I (via mevalonate) | 0.629 | -0.792 | NA |
| superpathway of glycerol degradation to 1,3-propanediol | -0.507 | 3.473 | 2.966 |
| superpathway of glycol metabolism and degradation | -1.911 | 4.578 | 2.667 |
| superpathway of glyoxylate bypass and TCA | -0.568 | NA | NA |
| superpathway of hexitol degradation (bacteria) | -1.444 | 1.087 | NA |
| superpathway of hexuronide and hexuronate degradation | -0.937 | 1.092 | NA |
| superpathway of L-arginine and L-ornithine degradation | -5.65 | 7.165 | 1.516 |
| superpathway of L-arginine, putrescine, and 4-aminobutanoate degradation | -5.65 | 7.165 | 1.516 |
| superpathway of L-aspartate and L-asparagine biosynthesis | 0.576 | NA | 0.74 |
| superpathway of L-threonine metabolism | -6.335 | 9.146 | 2.811 |
| superpathway of menaquinol-10 biosynthesis | -0.508 | NA | NA |
| superpathway of menaquinol-11 biosynthesis | -0.52 | NA | NA |
| superpathway of menaquinol-12 biosynthesis | -0.52 | NA | NA |
| superpathway of menaquinol-13 biosynthesis | -0.52 | NA | NA |
| superpathway of menaquinol-6 biosynthesis I | -0.508 | NA | NA |
| superpathway of menaquinol-8 biosynthesis II | 1.413 | -0.787 | 0.626 |
| superpathway of menaquinol-9 biosynthesis | -0.508 | NA | NA |
| superpathway of methylglyoxal degradation | -2.183 | 3.746 | 1.563 |
| superpathway of N-acetylglucosamine, N-acetylmannosamine and N-acetylneuraminate degradation | -0.896 | NA | -0.715 |
| superpathway of N-acetylneuraminate degradation | -0.663 | NA | -0.566 |
| superpathway of phenylethylamine degradation | -2.206 | 4.726 | 2.52 |
| superpathway of phylloquinol biosynthesis | -0.712 | NA | NA |
| superpathway of purine deoxyribonucleosides degradation | NA | 0.937 | 0.905 |
| superpathway of pyridoxal 5'-phosphate biosynthesis and salvage | NA | 2.341 | 2.37 |
| superpathway of pyrimidine deoxyribonucleosides degradation | NA | 0.648 | 0.768 |
| superpathway of salicylate degradation | -2.333 | 2.405 | NA |
| superpathway of sulfolactate degradation | NA | 1.819 | 1.324 |
| superpathway of sulfur oxidation (Acidianus ambivalens) | 1.533 | -1.735 | NA |
| superpathway of thiamin diphosphate biosynthesis II | 1.049 | -1.069 | NA |
| superpathway of UDP-glucose-derived O-antigen building blocks biosynthesis | -0.577 | 1.115 | 0.537 |
| superpathway of vanillin and vanillate degradation | -1.328 | 3.437 | 2.11 |
| TCA cycle VII (acetate-producers) | NA | 0.716 | NA |
| teichoic acid (poly-glycerol) biosynthesis | -1.136 | 4.14 | 3.004 |
| thiazole biosynthesis II (Bacillus) | 1.319 | -1.274 | NA |
| toluene degradation III (aerobic) (via p-cresol) | -2.388 | 2.255 | NA |
| toluene degradation IV (aerobic) (via catechol) | -2.366 | 2.877 | 0.511 |
| tRNA processing | NA | -0.591 | NA |
| UDP-2,3-diacetamido-2,3-dideoxy-&alpha;-D-mannuronate biosynthesis | 0.615 | -0.585 | NA |
| vanillin and vanillate degradation I | -1.328 | 3.437 | 2.11 |
| vanillin and vanillate degradation II | -1.325 | 3.426 | 2.101 |
| vitamin E biosynthesis (tocopherols) | -2.895 | 7.085 | 4.19 |
